# Plant Growth-Promoting Activity of Bacteria Isolated from Asian Rice (Oryza sativa L.) Depends on Rice Genotype

**DOI:** 10.1128/spectrum.02787-21

**Published:** 2022-07-11

**Authors:** Nasim Maghboli Balasjin, James S. Maki, Michael R. Schläppi, Christopher W. Marshall

**Affiliations:** a Marquette University, Biological Sciences Department, Milwaukee, Wisconsin, USA; Tufts University

**Keywords:** *Oryza sativa* L., plant growth, plant growth-promoting bacteria, phosphate-solubilizing bacteria

## Abstract

Asian rice is one of the most important crops because it is a staple food for almost half of the world’s population. To have production of rice keep pace with a growing world population, it is anticipated that the use of fertilizers will also need to increase, which may cause environmental damage through runoff impacts. An alternative strategy to increase crop yield is the use of plant growth-promoting bacteria. Thousands of microbial species can exist in association with plant roots and shoots, and some are critical to the plant’s survival. We isolated 140 bacteria from two distantly related rice accessions and investigated whether their impact on the growth of four different rice accessions. The bacterial isolates were screened for their ability to solubilize phosphate, a known plant growth-promoting characteristic, and 25 isolates were selected for further analysis. These 25 phosphate-solubilizing isolates were also able to produce other potentially growth-promoting factors. Five of the most promising bacterial isolates were chosen for whole-genome sequencing. Four of these bacteria, isolates related to Pseudomonas mosselii, a *Microvirga* sp., Paenibacillus rigui, and Paenibacillus graminis, improved root and shoot growth in a rice genotype-dependent manner. This indicates that while bacteria have several known plant growth-promoting functions, their effects on growth parameters are rice genotype dependent and suggest a close relationship between plants and their microbial partners.

**IMPORTANCE** In this study, endophytic bacterial isolates from roots and shoots of two distantly related rice accessions were characterized phenotypically and genotypically. From the isolated bacterial species, five of the most promising plant growth-promoting bacteria were selected to test their abilities to enhance growth of the four rice accessions. Interestingly, plant growth enhancement was both bacterial isolate specific and plant genotype specific. However, the positive interactions between plant and bacteria could not easily be predicted because rice growth-promoting bacteria isolated from their host plants did not necessarily stimulate growth of their own host.

## INTRODUCTION

Plant-microbe interactions are divided into three groups: pathogenic, symbiotic, and associative. Each of these interactions can affect plant physiology, such as nutrition level, growth and development, and defense mechanisms (1). These interactions can be beneficial to both plants and microorganisms (2). Plant-associated bacteria that benefit development of plants through direct and indirect mechanisms are known as plant growth-promoting bacteria (PGPB) (3). Direct mechanisms of plant growth promotion include phosphorus and zinc solubilization, nitrogen fixation, and plant hormone (phytohormone) production, and indirect mechanisms include antifungal activity, lytic enzyme production, siderophores, and ammonia production (3).

There are a variety of PGPB that inhabit the area near plant roots (rhizosphere), the root surface (rhizoplane), and inside the root (endosphere) (4). PGPB that live inside plant tissues are known as endophytes (5). Plants can attract specific bacteria from the soil environment to live on or inside their roots, to benefit the plants by providing and solubilizing nutrients, indicating the importance of the rhizoplane and the endosphere bacterial communities (6). On the other hand, plant roots provide carbon metabolites and nitrogen to bacteria (2). In addition, there are some bacteria that inhabit aerial parts of the plants known as the phyllosphere (7). Some of these phyllosphere bacteria are root endophytes that move through xylem vessels in the plant to eventually inhabit the aerial portions (8). Phyllosphere bacteria also benefit the plants through nitrogen fixation, plant hormone production, and biotic and abiotic stress tolerance (7). The most common plant growth-promoting bacteria belong to the genera *Bacillus*, Pseudomonas, Enterobacter, Acinetobacter, *Burkholderia*, *Arthrobacter*, and *Paenibacillus*. These bacteria are known to enhance plant immunity against pathogens and provide phytohormones, soluble phosphate, and/or nitrogen (8–11).

Asian rice (Oryza sativa L.) is one of the main staple foods for almost half of the world’s population (12). It is estimated that by 2050 food demand will be heightened due to an increasing global population (13). Therefore, the rate of fertilizer use will increase, causing environmental problems (14). One promising solution is to create environmentally friendly bio-fertilizers containing PGPB. Phosphorus is the second most important nutrient, after nitrogen, for plants but is typically insoluble in the soil (15). For plant roots to take up phosphate, insoluble inorganic phosphate needs to be converted to soluble phosphate (15, 16). Phosphate-solubilizing bacteria (PSB) play an important role because they secrete gluconic and keto-gluconic acids and phosphatases that release soluble phosphates into the soil (15–17). It has previously been shown that *Anabaena*, *Azospirillum*, *Rhodobacter*, and *Streptomyces* species can promote *O. sativa* growth, but further characterization is needed to expand the known species capable of growth promotion and to understand their mechanisms of action and their specificity for rice subspecies (4, 15, 18). Therefore, discovering and characterizing PSB can improve crop productivity with less environmental impact than traditional fertilizers.

In this study, we screened for PGPB by isolating bacteria from the surface and inner parts of leaves and roots of two distantly related rice varieties belonging to the two subspecies of *O. sativa*, *indica* and *japonica*. Bacterial isolates were evaluated for plant growth-promoting phenotypes, and the genomes of five PGPB were sequenced. Finally, we separately evaluated the effect of the sequenced isolates on growth and development of the two rice accessions from which they were isolated. In addition to these two rice accessions, we evaluated the influence of the isolated PGPB on two other rice accessions, to understand whether different rice accessions respond differently to PGPB in a matter of growth and development. To the best of our knowledge, this is the first work evaluating the influence of *O. sativa* endophytes, which were isolated from both *indica* and *japonica*, on the growth and development of members of each of the two subspecies. The endophytes were isolated from two separate varieties of two rice subspecies but were tested on 4 total rice varieties, 2 each of the different subspecies.

## RESULTS

### Bacterial isolation and characterization.

**FIG 1:**
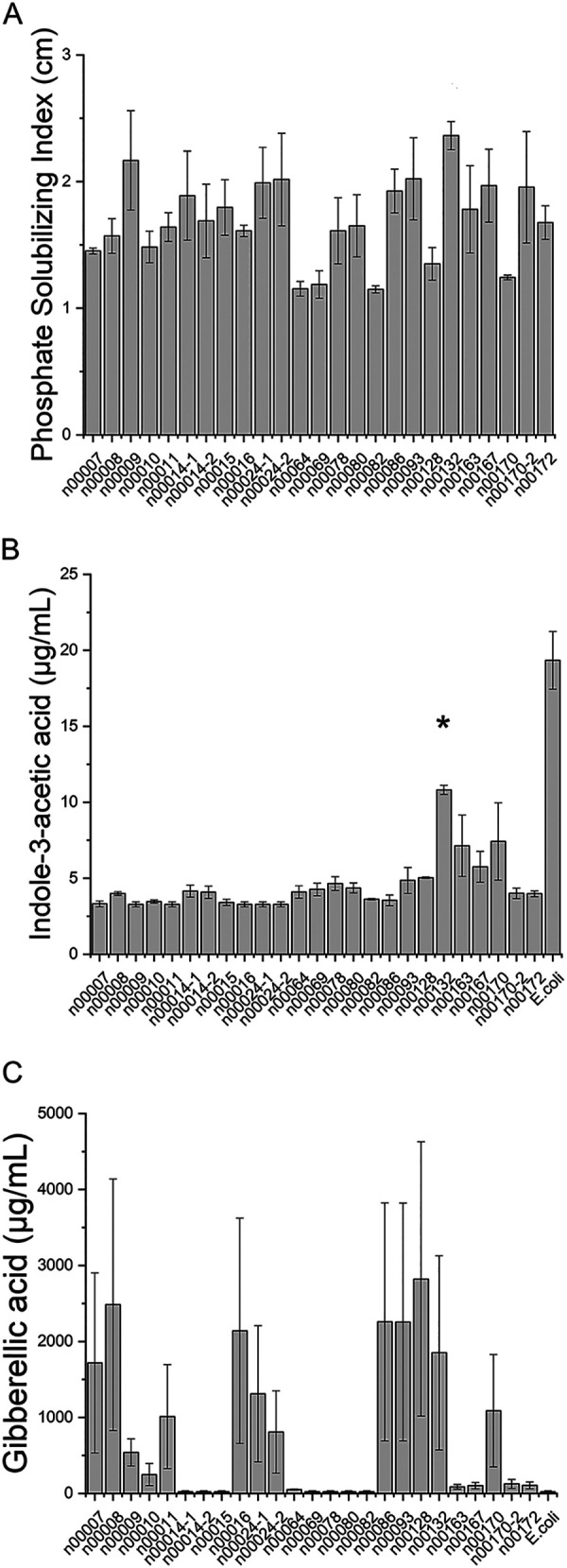
(A) Mean phosphate solubilization index of three trials. Phosphate solubilization activity was evaluated by the measurement of halo diameters around bacterial colonies (in cm) relative to colony diameter on NBRIP (National Botanical Research Institute’s Phosphate) medium. There was no significant difference in the solubilization index of all 25 PSB (*P > *0.05, ANOVA). (B) Indoleacetic acid (IAA) production by phosphate-solubilizing bacteria (PSB). E. coli strain OP50 was used as the positive control for IAA production. n00132 produced a significantly larger amount of IAA than other PSB isolates, except for n00170 (*P < *0.05, ANOVA and Tukey *post hoc* test; E. coli was not used in statistical analysis). (C) Gibberellic acid (GA) production by PSB. E. coli strain OP50 was used as the negative control for GA production. PSB isolates n00014-1, n00014-2, n00015, n00064, n00069, n00078, and n00080 were not able to produce GA, as they were not different from the negative control. There was no difference in GA production among the 18 bacteria (*P > *0.05, ANOVA). Error bars show standard errors.

To identify plant growth-promoting bacteria (PGPB) associated with rice plants, a total of 140 bacteria were isolated and purified from the phyllosphere, root endosphere, and root rhizoplane of two accessions representing the two *japonica* and *indica* subspecies of rice (see Table S4 in the supplemental material). The colony morphology and Gram reaction of each isolated bacterium are listed in Table S5. Because phosphate is the second most important macronutrient for plant growth (17), we reasoned that mineral phosphate-solubilizing bacteria (PSB) associated with rice tissues might act as PGPB. Phosphate is mostly insoluble in the soil and therefore unavailable to plants (19). There are PSB in the soil with the ability of providing soluble phosphate through releasing organic acids and acid phosphatase (20). To identify PSB among the 140 isolates, bacteria were spotted onto agar plates containing insoluble phosphate [Ca_3_(PO_4_)_2_]. Of the 140 isolates, 25 (18%) were able to solubilize mineral phosphate as shown by a halo around the bacterial colonies. The mean phosphate solubilization index was calculated for those 25 bacteria (Fig. 1A). There was no significant difference in the solubilization index of all 25 PSB (*P > *0.05, analysis of variance [ANOVA]) (Fig. S3C).

We next characterized these 25 phosphate-solubilizing isolates for other possible plant growth-promoting traits, including nutrient sequestration and hormone production (3). Of the 25 PSB isolates, 17 (68%) were considered nitrogen-fixing microorganisms that could also contribute to plant growth (Fig. S3E and Table S3). Only one isolate (n00078) was able to solubilize zinc sulfate (Fig. S3D). Of the phytohormones tested, all 25 PSB isolates had the ability of indoleacetic acid (IAA) production, and among them, isolate n00132 produced the largest amount of IAA (10.8 μg mL^−1^; *P < *0.05, ANOVA, Tukey *post hoc* test), two to three times more than the other isolates (Fig. 1B). Gibberellic acid (GA) production was observed in 72% (18/25) of the isolates (Fig. 1C). There was no difference in GA production among the 18 bacteria (*P > *0.05, ANOVA).

High soil salinity negatively affects the ability of plant roots to take up water, and bacteria that can tolerate high concentrations of NaCl were previously shown to protect plants from water deficiency stress (21). Therefore, all 25 PSB isolates were tested for salt tolerance on LB medium with different concentrations of NaCl (2%, 4%, 6%, and 8%). All 25 PSB were able to tolerate at least 2% NaCl, and 80% (20/25) of them grew on LB with 4% NaCl (Table S6). Only one bacterium (n00014-1) grew on LB with 6% NaCl, and none of the PSB were able to tolerate 8% NaCl.

Ethylene is a phytohormone that plant roots produce for developmental processes such as xylem formation and to regulate stress responses (22). When plants are under stress, they often emit excessive ethylene, which has a negative effect on plant development. Bacteria that produce 1-aminocyclopropane-1-carboxylic acid (ACC) deaminase convert ACC, a rate-limiting precursor for ethylene production, to ammonia and α-ketobutyrate, which can protect plants from excessive amounts of ethylene (23). In this study, PSB that produced ACC deaminase enzyme were examined based on their ability to grow on Dworkin & Foster (DF) minimal salt medium supplemented with 3 mM ACC as the sole nitrogen source (Fig. S3F and Table S7). A total of 84% (21/25) of the isolates had ACC deaminase activity that might protect plants from excessive ethylene buildup (Table S7).

PSB isolates were next tested for antifungal activity, lipase production, protease activity (casein and gelatin hydrolyzing), and cellulase and ammonia production, all of which can contribute to bacterial defense mechanisms against plant pathogens (24). PSB isolates were also tested for siderophore production, which is a low-molecular-mass compound with high affinity to Fe^3+^ and facilitates iron uptake by plants (25). The results of these indirect PGPB assays are summarized in Table S6. Based on the results, 68% (17/25) of the PSB isolates were able to facilitate iron uptake in plants and produce lipase enzyme, and 84% (21/25) of the PSB were able to produce cellulase enzymes. In total, 56% (14/25) of the phosphate-solubilizing isolates were able to liquify gelatin, among which 71% (10/14) strongly liquified it and 29% (4/14) slightly liquified it. Only 8% (2/25) of the phosphate-solubilizing isolates were able to hydrolyze casein, and 84% (21/25) of the PSB isolates were able to inhibit fungal pathogen growth. All 25 PSB isolates were able to produce ammonia. These results showed that all 25 bacterial isolates tested had some ability to indirectly improve plant growth through pathogen control.

### Whole-genome sequencing and phenotypic analysis of five PSB.

**FIG 2:**
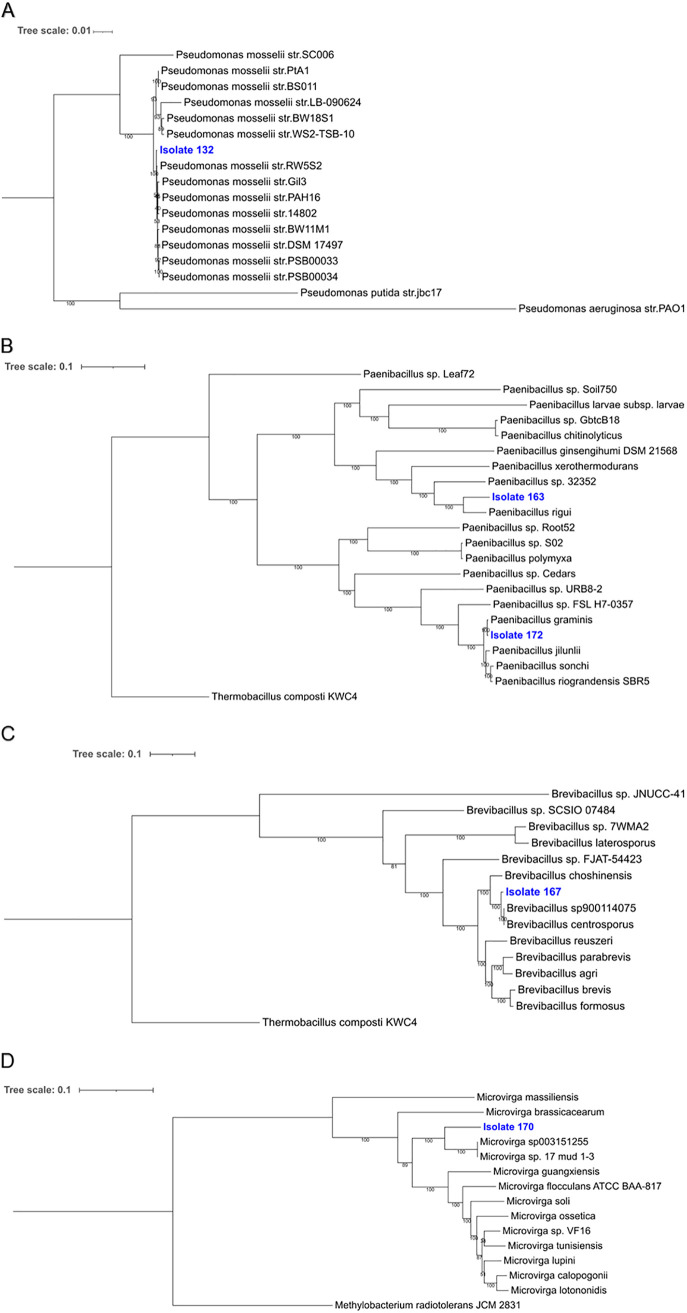
Maximum likelihood phylogenetic trees based on whole-genome sequences of five bacteria isolated from different rice tissue compartments. The tree scale is the number of nucleotide substitutions per site. (A) Pseudomonas mosselii, isolate 132, from *indica* leaves. (B) Paenibacillus rigui, isolate 163, from *indica* endosphere and Paenibacillus graminis, isolate 172, from *japonica* leaves. (C) Brevibacillus sp900114075, isolate 167, from *indica* leaves. (D) Microvirga sp003151255, isolate 170, from *japonica* leaves.

Five bacterial isolates that were positive for phosphate solubilization, IAA, and GA production and nitrogen fixation were selected for whole-genome sequencing. The genome assembly statistics and taxonomic assignments for the five isolates are shown in Table 1. Three of the isolates (n00163, n00167, and n00172) were members of the Gram-positive *Paenibacillaceae* family, while the others belonged to the Gram-negative *Proteobacteria* phylum (Fig. 2). Three of the five isolates (n00163, n00167, and n00170) fell near or below the 95% average nucleotide identity (ANI) threshold that is sometimes used for species delineation (26–28), indicating that these isolates are potentially new species. The other two isolates were new strains of Pseudomonas mosselii and Paenibacillus graminis (Table 1). All five of the isolates had genes predicted for synthesis of the phytohormone auxin (IAA), for ammonia assimilation, and for phosphate metabolism. These genome annotations provide the genotypes to accompany their plant growth-promoting phenotypes.

**TABLE 1:** Feature types and assembled reads with SPAdes v3.13.0 results (QUAST analysis) of sequenced genomes and Genome Taxonomy Database (GTDB) analysis results of PSB isolated from different rice subspecies and tissues

Characteristic	Data for strain:				
	n00163	n00167	n00132	n00172	n00170
Genome size (bp)	7,304,039	6,449,936	5,782,008	7,161,689	4,587,927
G+C ratio (%)	52	49	64	50	63
No. of contigs	89	153	133	104	46
*N*_50_ (bp)	172,653	100,283	76,736	161,901	235,294
No. of predicted genes	6,856	6,759	5,305	6,966	4,557
Rice subspecies & tissue compartment	*indica* endosphere	*indica* leaves	*indica* leaves	*japonica* leaves	*japonica* leaves
Avg nucleotide identity (%)	80.61	95.39	99.23	98.41	82.89
Closest genome	Paenibacillus rigui	*Brevibacillus* sp900114075	Pseudomonas mosselii	Paenibacillus graminis	*Microvirga* sp003151255

Gen III microplates (Biolog) were used to characterize the metabolic phenotypes of the five sequenced PSB. Agrobacterium tumefaciens, a Gram-negative plant pathogen producing crown gall tumors (29), was used as a comparison because of its well-characterized ability to use plant metabolites as nutrients (30). Of the five isolates tested, two (n00163 and n00167) shared similar phenotypic carbon source usage characteristics (Table S8). Of the different metabolic phenotypes, methyl pyruvate is a plant chemoeffector (31) and glycerol is a leaf exudate as a result of carbon dioxide fixation in photosynthesis (32). Glycerol was not used by n00132, while methyl pyruvate was not used by n00132 and n00172. Interestingly, n00172 was the only bacterium able to utilize stachyose (raffinose family of oligosaccharides in plants) (33). All five sequenced PSB isolates shared the same phenotypic chemical resistance against pH 6, 1% NaCl, 1% sodium lactate, guanidine HCl, tetrazolium blue, nalidixic acid, lithium chloride, potassium tellurite, and aztreonam. Only one bacterium (n00172) showed resistance against rifamycin SV and sodium butyrate (Table S9).

### Antibacterial activity of the sequenced PSB against each other.

Overlay-agar and cross-streak assays were done to determine whether the five sequenced PSB were compatible with each other. Plants derive greater benefits from the mixture (consortium) of PGPB than one bacterium (34). Therefore, it is important to investigate whether these five PSB would compete, act neutrally, or act synergistically to improve rice growth. Based on these assays, only isolate n00172 competitively inhibited the growth of the other four PSB (Fig. S3A and B). This indicates that n00172 (Paenibacillus graminis) produced inhibitory chemicals that did not allow other bacteria to grow in its close proximity (Table S10). Analysis of the n00172 genome through the program antiSMASH (35) revealed that this bacterium has several biosynthetic gene clusters that are predicted to have antibiotic activity (Table S11) that may be responsible for competitive inhibition of other species.

### Influence of sequenced PSB on rice plant growth and development.

**FIG 3:**
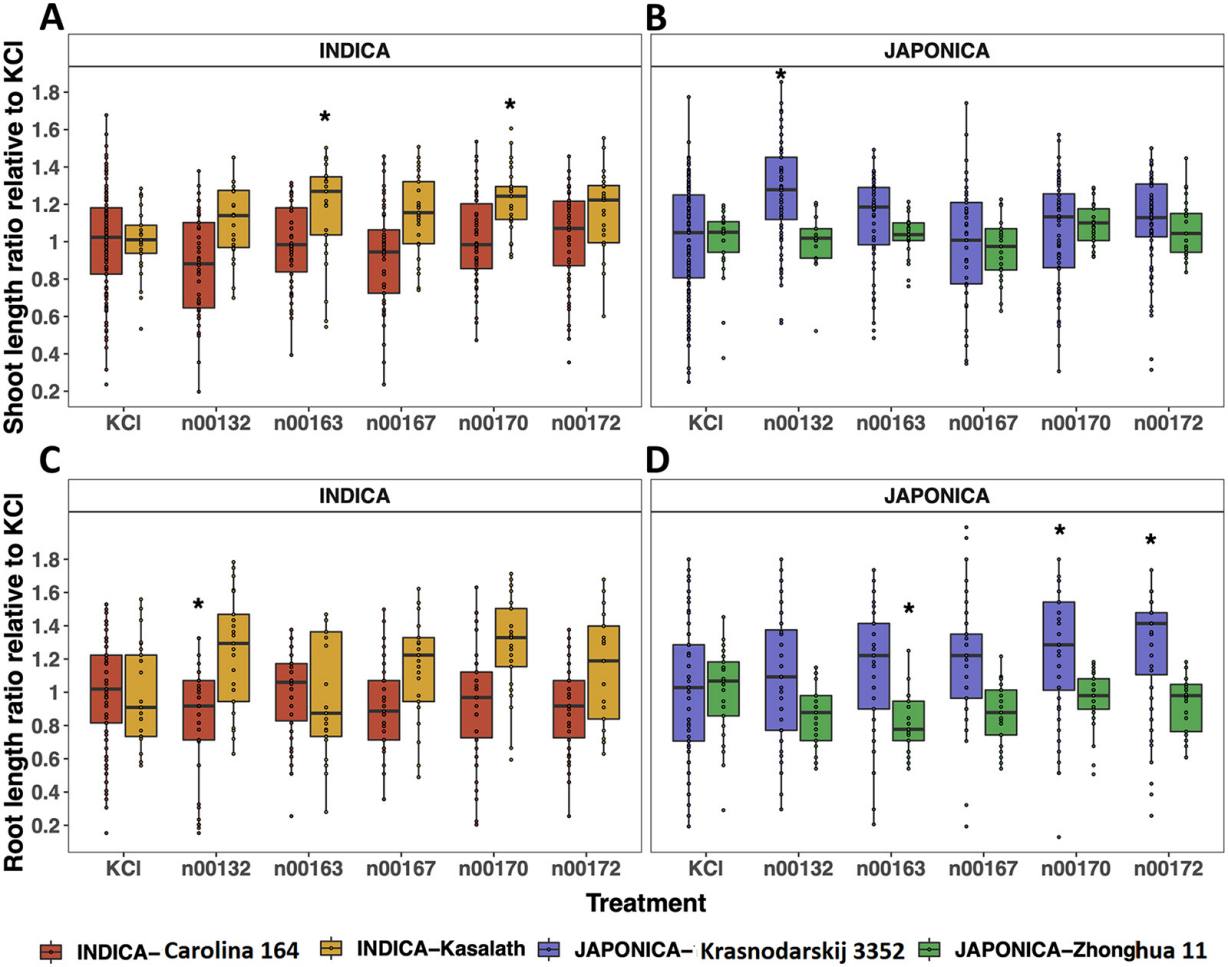
(A to D) Relative shoot (A and B) and root (C and D) growth of the 2-week-old treated plants with five bacterial species compared to shoot and root growth of the uninoculated control plants (KCl). (A and B) Shoot length ratio relative to control (KCl) plants belonging to *indica* and *japonica* subspecies: *indica* Carolina 164, *indica* Kasalath, *japonica* Krasnodarskij 3352, and *japonica* Zhonghua 11. (C and D) Root length ratio relative to control (KCl) plants belonging to *indica* and *japonica* subspecies: *indica* Carolina 164, *indica* Kasalath, *japonica* Krasnodarskij 3352, and *japonica* Zhonghua 11. Each dot represents an individual plant. Statistical significance was determined by Kruskal-Wallis and Dunnett post hoc tests. *, *P < *0.05.

To determine whether the five sequenced PSB act as PGPB, seeds from the four rice varieties, representing the *indica* and *japonica* subspecies, were inoculated individually with one of the five PSB, and the shoot and root development (length) and biomass (dry weight) of 2-week-old plants were compared to those of uninoculated control plants (Fig. 3). Control plants were from uninoculated seeds that were soaked in KCl solution without any bacteria. *O. sativa* subsp. *indica* Kasalath varietal plants inoculated with n00163 (Paenibacillus rigui) and n00170 (*Microvirga* sp.) had significantly higher relative shoot growth than that of Kasalath control plants (Fig. 3A; *P < *0.05, Kruskal-Wallis, Dunnett *post hoc* test). There was no shoot growth promotion observed in *indica* Carolino 164 varietal plants inoculated with the five bacteria. On the other hand, *japonica* Kasnodarskij 3352 varietal plants inoculated with n00132 (Pseudomonas mosselii) had significantly higher relative shoot growth than that of Kasnodarskij 3352 control plants (Fig. 3B; *P < *0.05, Kruskal-Wallis, Dunnett *post hoc* test). Similar to *indica* Carolino 164 varietal plants, no shoot growth promotion was observed in *japonica* Zhonghua 11 varietal plants inoculated with the five bacteria. In addition to relative shoot length, *indica* Kasalath and *japonica* Zhonghua 11 varietal plants inoculated with n00132 (Pseudomonas mosselii) and n00163 (Paenibacillus rigui), respectively, responded negatively in relative root length ratio compared to their control varietal plants (Fig. 3C and D; *P < *0.05, Kruskal-Wallis, Dunnett *post hoc* test). On the other hand, *japonica* Kasnodarskij 3352 varietal plants inoculated with n00170 (*Microvirga* sp.) and n00172 (Paenibacillus graminis) had significantly increased root growth compared to that of Kasnodarskij 3352 control plants (Fig. 3D; *P < *0.05, Kruskal-Wallis, Dunnett *post hoc* test). Taken together, these findings showed that the four bacterial species, n00132 (Pseudomonas mosselii), n00163 (Paenibacillus rigui), n00170 (*Microvirga* sp.), and n00172 (Paenibacillus graminis), were PGPB for the *japonica* Kasnodarskij 3352 and *indica* Kasalath varietal plants for the characteristics tested here, while none of the five species were PGPB for the *japonica* Zhonghua 11 or *indica* Carolino 164 varietal plants of rice.

**FIG 4:**
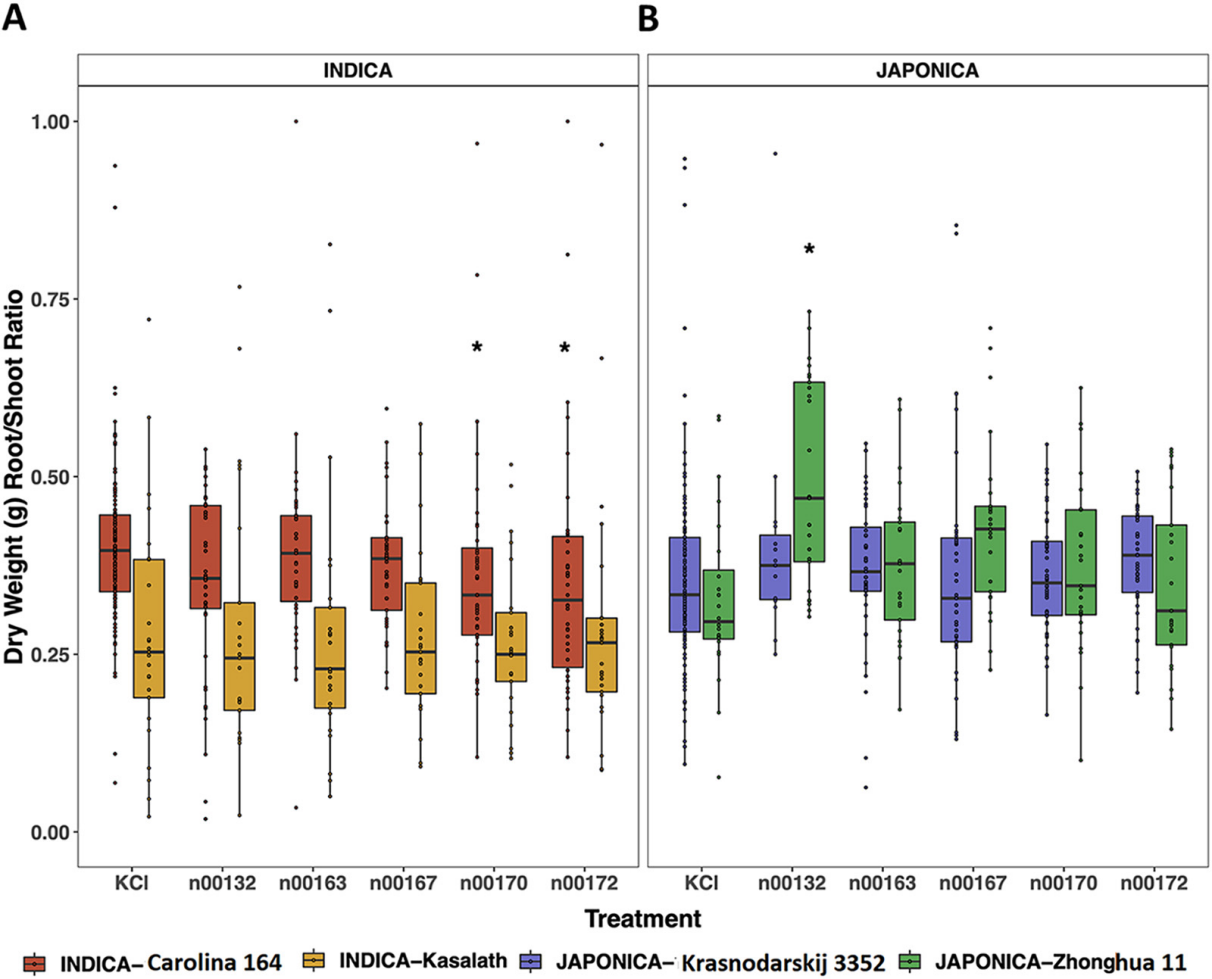
Dry weight root/shoot ratios of the 2-week-old treated plants with five bacterial species compared to uninoculated control plants (KCl). (A) Root/shoot ratios of the two *indica* varieties: *indica* Carolina 164 and *indica* Kasalath. (B) Root/shoot ratios of the two *japonica* varieties: *japonica* Krasnodarskij 3352 and *japonica* Zhonghua 11. Each dot represents an individual plant, and outliers (values of >1) were omitted from the plot. Statistical significance was determined by Kruskal-Wallis and Dunnett post hoc tests. *, *P < *0.05.

Root/shoot ratio refers to the biomass that is growing underground and reflects the ability of plants to take up nutrients. The rationale for assessing this is that the higher this ratio, the greater the ability of plants to take up nutrients, which correlates with increased stress tolerance (36). Our measurements showed that *indica* Carolino 164 inoculated with n00170 (*Microvirga* sp.) and n00172 (Paenibacillus graminis) had lower dry weight root/shoot ratios (*P < *0.05, Kruskal-Wallis, Dunnett *post hoc* test), suggesting that these two species do not help with nutrient uptake (Fig. 4A). Moreover, the *indica* Kasalath and *japonica* Krasnodarskij 3352 varietal plants inoculated with the five bacteria did not have significantly improved root/shoot ratios compared to uninoculated control plants (Fig. 4A and B). In contrast, only the *japonica* Zhonghua 11 varietal plants inoculated with n00132 (Pseudomonas mosselii) had an improved dry weight root/shoot ratio compared to control plants (Fig. 4B; *P < *0.05, Kruskal-Wallis, Dunnett *post hoc* test). Taken together these findings show that only Pseudomonas mosselii helped the *japonica* Zhonghua 11 varietal plants with nutrient uptake.

## DISCUSSION

To identify plant growth-promoting bacteria (PGPB) from rice, we isolated 140 bacteria residing in or on different tissues of two distantly related rice varieties and screened for phosphate-solubilizing activity. It is worth noting that the Asian rice species (*O. sativa* L.) is divided into two subspecies, long-grain *indica* and short-grain *japonica*, both of which contain several subgroups. The *indica* subspecies contains the *aus* and *indica* subgroups, and the *japonica* subspecies contains the tropical *japonica*, temperate *japonica*, and aromatic subgroups. In this study, bacteria were isolated from *aus* and temperate *japonica* accessions Carolina 164 and Krasnodarskij 3352, respectively, because they represent subgroups with major genotypic and phenotypic differences (37). The bacteria isolated from these two varietal groups of rice were tested for their plant growth-promoting influence on the rice variety that they were isolated from and the other three rice varieties listed above.

The vegetative growth stage in rice plants, which encompasses seed germination to stem elongation, is a critical stage for the formation of root exudates (38) and interactions with bacteria. Large amounts and different types of carbohydrates and organic acids are produced by roots at this stage (38, 39). Therefore, we chose to work with young seedlings (when rice plants newly develop the second leaf) to observe the interaction between plants and bacteria, as this is when rice plants start to produce different metabolites that can provide bacteria with nutrients.

Of the 140 isolates, 25 were phosphate-solubilizing bacteria (PSB) that were also able to produce IAA, an important plant-growth hormone. Based on other direct and indirect PGPB assays, five PSB were selected for whole-genome sequencing. The five PSB species were closely related to Paenibacillus rigui (n00163, isolated from the Carolino 164 endosphere), *Brevibacillus* spp. (n00167, isolated from Krasnodarskij 3352 leaves), Pseudomonas mosselii (n00132, isolated from Carolino 164 leaves), Paenibacillus graminis (n00172, isolated from Krasnodarskij 3352 leaves), and *Microvirga* spp. (n00170, isolated from Krasnodarskij 3352 leaves).

Although only four of the PSB improved growth and development of the two *japonica* varieties and the *indica* Kasalath variety tested here, all of these bacterial genera were previously shown to function as PGPB (29, 40–43). There are some *Brevibacillus* species that are able to produce antifungal siderophores (42). The *Microvirga* genus is a nodule legume endophyte that can improve plant growth by providing ammonia to plants (44). Some species of *Paenibacillus* have been isolated from plant rhizospheres, and *P*. *graminis* is a known nitrogen-fixing bacterium (28, 45). *P. rigui* is one of the species that has been specifically isolated as an endophyte from *O. sativa* (46). Pseudomonas species are widely present in soils, and it is known that some strains can survive under different environmental conditions and tissues of eukaryotic hosts mostly through producing chemicals that protect them against pathogenic bacteria or fungi (29, 43). *P*. *mosselii* was previously shown to be a phosphate-solubilizing bacterium (47), and our results are in agreement with this finding (Fig. 1A). *P*. *mosselii* is also one of the most common PGPB that inhibits growth of plant pathogens such as Agrobacterium tumefaciens (29), and results from our study (cross-streak experiment) confirm this observation (Fig. S4). In addition, *P. mosselii* is also known for improving plant growth and increasing sugar content of Agave americana L. (43), and our results are in agreement with some of these findings (Fig. 3 and 4). Therefore, this bacterium is a good candidate for promoting plant growth and potentially also for protecting plants against stress (48).

Auxins such as IAA are an important class of plant-growth hormones (phytohormones) (49), and auxin-producing PSB might be PGPB by stimulating root growth and differentiation. The 25 PSB isolates were tested for IAA production, which was previously shown to act as a plant growth-promoting phytohormone when produced by plant-associated bacteria (42, 50). Our results specifically showed that n00132 (*P. mosselii*) was the bacterium with a higher (not significant) phosphate solubilizing index, (2.36 cm; Fig. 1A) and significantly highest IAA production (10.81 μg mL^−1^; Fig. 1B). In addition, this bacterium was the only one with positive results for all indirect PGPB pathways, including siderophore, cellulase, lipase, protease, and ammonia production.

Among all five bacterial species, the *Brevibacillus* sp. was the only one that did not show positive effects on growth and development of the four rice varieties tested here, despite the fact that this bacterium is considered a PGPB with the positive *in vitro* PGPB assays of this study. According to Rapid Annotations using Subsystems Technology (RAST) genomic analysis (51), the *Brevibacillus* sp. isolated from the *indica* Carolina 164 variety does not contain sequences for denitrifying reductase gene clusters, and this might be one of the reasons why this bacterium had no effect on growth and development of the four rice varieties tested here. Without denitrifying reductase gene clusters, root formation and development in plants will not be efficient (52).

Interestingly, the results of this study showed that four sequenced bacteria were able to increase root and/or shoot growth and root or shoot biomass of 2-week-old *japonica* Krasnodarskij 3352, *japonica* Zhonghua 11, and *indica* Kasalath varietal plants but had no (all five bacteria) or a negative (*P. mosselii* [n00132]) effect on the growth of 2-week-old *indica* Carolino 164 varietal plants tested here. Furthermore, it is interesting to note that *P. mosselii* isolated from the leaf of the *indica* Carolino 164 variety had a positive shoot growth-promoting effect on the *japonica* Krasnodarskij 3352 varietal plants but a negative effect on root growth of the rice variety it was isolated from (*indica* Carolina 164). More interestingly, the other bacterium (*P. rigui* [n00163]) isolated from the root of the *indica* Carolino 164 variety had a negative effect on root growth of the *japonica* Zhonghua 11 variety but a positive effect on shoot growth of the *indica* Kasalath variety. The *Microvirga* sp. bacterium (n00170) was isolated from the leaf of Krasnodarskij 3352 and improved shoot growth of the *indica* Kasalath rice variety. Both the *Microvirga* sp. and *P. graminis* (n00172) isolated from the leaf of Krasnodarskij 3352 improved root growth of the rice variety they were isolated from. For nutrient uptake ability, *P. mosselii* had a positive effect on the *japonica* Zhonghua 11 variety, while the *Microvirga* sp. and *P. graminis* had negative effects on the *indica* Carolina 164 variety. These results reveal a distinct bacterial behavior when applied to different rice varieties. That is, some bacterial species were compatible with the rice variety they were isolated from and improved growth and development of the local rice variety, while other bacterial species improved growth and development of nonlocal rice varieties.

To the best of our knowledge, this is the first work describing a positive plant growth-promoting effect of PSB residing in two distantly related rice accessions on only one of the varieties they were isolated from as well as other rice varieties. There are at least two possibilities for this observation. First, genetic differences between the two varieties of *japonica* and the two varieties of *indica* plants assayed might lead to differential responses to bacterial metabolism (53). It is reasonable to assume that PSB interact with and possibly regulate plant genes for growth and development. These genes could be differentially expressed in the four rice varieties or absent in one or two of the varieties. Second, there might be differences in how genes/operons and/or metabolic pathways within the five PSB respond to different exudates and plant hormones. Further studies need be done to understand the mechanisms of how these bacteria respond differently to different rice varieties, and *vice versa*, how different rice varieties respond differently to these bacteria. Additionally, we are interested in understanding the longer-term effects of PSB on plants, as the present work focused on the critical period of growth in the first 2 weeks. While many studies have discovered the benefits of PGPB (3, 15, 46, 53, 54), we demonstrate here that the benefits may not be universally applied. Even plant taxa as closely related as subspecies will respond differently to PGPB, indicating the need to develop strain specificities between plants and bacteria.

## MATERIALS AND METHODS

### Plant growth and bacterial isolation from roots and leaves.

We used accessions from the two subspecies of *O. sativa*: Krasnodarskij 3352 (Genetic Stock Oryza [GSOR] no. 311787) and Zhonghua 11 from the temperate *japonica* subpopulation representing the *japonica* subspecies and Carolino 164 (GSOR no. 311654) and Kasalath (GSOR no. 301077) from the *aus* subpopulation representing the *indica* subspecies. Rice seeds were obtained from the Dale Bumpers National Rice Research Center (DBNRRC), Stuttgart, AR. Carolino 164 and Kasalath seeds were from DBNRRI field-grown plants, while Krasnodarskij 3352 seeds were from plants grown in raised-bed roof-top paddies at Marquette University in Milwaukee, WI, and Zhonghua 11 seeds were from plants grown at the Chinese Academy of Sciences, Beijing, China.

Following the methods of Edwards et al. (4), rice seeds were surface-sterilized with minor modifications. Briefly, seeds were dehulled and immersed in ethanol (70% vol/vol in distilled water [dH_2_O]) for 1 min, rinsed with sterile water (3 times), immersed in sodium hypochlorite (70% vol/vol in dH_2_O) for 5 min, and rinsed again with sterile water (3 times). After surface sterilized seeds were dried on autoclaved filter papers, seeds were put into sterile magenta boxes containing agar-solidified Murashige-Skoog (MS) medium (4) for germination and incubated at 37°C (2 days). Germinated seeds on MS medium were transferred to a growth chamber with cycles of 12 h of light (28°C) and 12 h of dark (24°C) for 10 days. Then, seedlings were transplanted into soil pots and incubated in the same growth chamber. Soil pots contained a 50:50 mixture of two commercial soil mixes. The nutrient contents of each soil mix are listed in Table S1.

Isolation of bacteria from the roots (rhizoplane and endosphere) and leaves (phyllosphere) during vegetative growth (when plants were 2 weeks old) was done as described previously (55). Isolation of bacteria from roots and shoots was done with the Krasnodarskij 3352 accession from the *japonica* subspecies and the Carolino 164 accession from the *indica* subspecies. Roots and leaves of both rice subspecies were rinsed with a sterile phosphate-buffered saline (PBS; 8 g NaCl, 0.2 g KCl, 1.44 g Na_2_HPO_4_, 0.24 g KH_2_PO_4_, pH = 7.4 with HCl; total volume = 1 L) to remove the attached soil from roots and other contaminants from leaves. Rinsed roots and leaves were sonicated 3 times in sterile PBS and surface-sterilized following the methods of reference 40. Briefly, the roots and leaves were rinsed with sterile water and then soaked in ethanol (75% vol/vol in dH_2_O) for 1 min and again rinsed with sterile water. Next, they were soaked in sodium hypochlorite (1% vol/vol in dH_2_O) for 1 min and finally washed with sterile water. Sterile roots and leaves were crushed in PBS with a sterile mortar and pestle. The PBS that contained crushed roots and leaves was transferred into 1.5-mL sterile tubes and centrifuged (12,074 × *g*, 10 min). Then, 100 μL of the supernatant was plated on nutrient agar (NA) medium (Difco) and incubated at 30°C. Cultures were purified by repeatedly streaking bacteria onto NA and incubating them at 30°C until only one colony type remained.

### Colony and cell morphology of isolated bacteria from roots and leaves.

The colony and cell morphology of isolated bacteria were determined through observing purified cultures and Gram staining, respectively. Colony morphology characteristics, including form, elevation, size, color, and margin were investigated (https://laboratoryinfo.com/colony-morphology-of-bacteria/, accessed during the spring of 2019).

Pure cultures (optical density at 600 nm [OD_600_] = 0.6) used for agar assay methods and spot inoculations were grown in nutrient broth (NB) medium at 30°C for 24 h.

### Mineral phosphate solubilization activity test.

Pure cultures were grown in nutrient broth (NB) medium at 30°C for 24 h. An agar assay method was used to screen the phosphate-solubilizing ability of bacterial isolates. Following the methods of reference 56, the National Botanical Research Institute’s phosphate (NBRIP) growth medium containing glucose (10 g L^−1^), Ca_3_(PO_4_)_2_ (5 g L^−1^), MgCl_2_·6H2O (5 g L^−1^), MgSO_4_·7H_2_O (0.25 g L^−1^), KCl (0.2 g L^−1^), and (NH_4_)_2_SO_4_ (0.1 g L^−1^) was used for this approach. First, 2 μL of each isolated bacterium (OD_600_ = 0.6) was spot inoculated onto NBRIP medium in three replicates. Plates were incubated at room temperature for 14 days. After 2 weeks, plates were examined for phosphate solubilization activity as shown by a clear halo around the colony. The phosphate solubilization index (56) was calculated from three independent experiments as follows:

$$\text{Phosphate solubilizing index}=\frac{\text{colony diameter }\left(\text{cm}\right)\;+\text{ halo diameter }\left(\text{cm}\right)}{\text{colony diameter }\left(\text{cm}\right)}$$

### Screening PSB for other plant growth-promoting features.

Both direct and indirect mechanisms of plant growth-promoting screening tests were done on PSB using the following culture-based assays (also listed in Table S2).

**Zinc solubilization.** Tris minimal medium (Tris-HCl [6.06 g L^−1^], NaCl [4.68 g L^−1^], KCl [1.49 g L^−1^], NH_4_Cl [1.07 g L^−1^], Na_2_SO_4_ [0.43 g L^−1^], MgCl_2_·2H_2_O [0.2 g L^−1^], CaCl_2_·2H_2_O [30 mg L^−1^], and agar [15 g L^−1^]) with 0.1% (wt/vol in distilled water) zinc sulfate (ZnSO_4_) was prepared, and 2 μL of each isolated strain (OD_600_ = 0.6) in PBS was spot-inoculated onto the above-described medium in three replicates. Plates were incubated at 30°C for 14 days. After 2 weeks, plates were examined for zinc solubilization activity. Similar to phosphate solubilization, a halo zone around each bacterium was confirmation for zinc solubilization (57). This experiment was repeated twice, independently.

**Nitrogen fixation.** For confirmation of nitrogen fixation, through culture-based assays, 2 μL of PSB (OD_600_ = 0.6) was spot-inoculated onto nitrogen-rich yeast extract-mannitol agar containing Congo red dye (CR-YMA) and incubated at 30°C for 5 days. Weak absorption of Congo red dye by colonies was confirmation for nitrogen fixation. Bacterial colonies with colors ranging from white to pale white-pink were able to fix nitrogen (58) due to cleavage of the azo bond (–N = N–) in Congo red (59). This experiment was repeated three times, independently. *Rhizobium* spp. and Staphylococcus aureus were used as positive and negative controls, respectively.

**Indoleacetic acid (IAA) production.** A colorimetric assay was done using the ferric chloride-perchloric acid reagent (FeCl_3_-HClO_4_) to detect IAA production. Indole compounds were quantified in precursor l-tryptophan medium. Nutrient broth-M26 (NaCl 5 [g L^−1^], peptone [10 g L^−1^], and beef extract [10 g L^−1^]) was used to grow bacteria for 24 h on a shaker (150 rpm) at 28°C. Then, 100 μL of the culture was inoculated into 10 mL of liquid minimal salt medium (KH_2_PO_4_ [1.36 g/L], Na_2_HPO_4_ [2.13 g L^−1^], MgSO_4_·7H_2_O 0.2 g L^−1^], and trace elements [containing citric acid (5 g/100 mL), ZnSO_4_·7H_2_O (5 g/100 mL), FeSO_4_·7H_2_O (4.75 g/100 mL), Fe(NH_4_)2(SO_4_)2·6H_2_O (1 g/100 mL), CuSO_4_·5H_2_O (250 mg/100 mL), MnSO_4_·H_2_O (50 mg/100 mL), H_3_BO_3_ (50 mg/100 mL), and Na_2_MoO_4_·2H_2_O (50 mg/100 mL) in 100 mL distilled water] [1 mL]) supplemented with 5 mM l-tryptophan and incubated at 28°C for 48 h on a shaker (150 rpm). l-tryptophan medium contained glucose (10 g L^−1^), l-tryptophan (1 g L^−1^), and yeast extract (0.1 g L^−1^) in 100 mL water and was filter-sterilized through a 0.2-μm membrane (Whatman syringe filters). After 48 h of growth, 1.5 mL of bacterial solution was centrifuged at 8,870 × *g* for 5 min in a microcentrifuge. Then, 1 mL of the supernatant was mixed with 2 mL FeCl_3_-HClO_4_ reagent, and after 25 min, the mixture’s optical density (OD) was measured using a UV-spectrophotometer at 530 nm. A standard curve (with concentrations of 0 to 300 μg/mL) was created for calculating the micrograms of IAA per milliliter of the mixture (60). This experiment was repeated twice, independently.

**Gibberellic acid (GA) production.** GAs are other important phytohormones regulating plant growth, seed germination, and stem elongation (54). Following the methods of references 54 and 49, the GA production detection assay was done with minor modifications. Briefly, PSB were freshly grown in NB (Difco) medium for 24 h at 30°C and then incubated at room temperature for 1 week. After growth, 1.5 mL of bacterial suspensions was centrifuged (3,942 × *g*) for 10 min in a microcentrifuge. Then, 1 mL of supernatant was transferred into 15-mL tubes, and 2 mL of zinc acetate solution (zinc acetate [21.9 g L^−1^], glacial acetic acid [1 mL], and distilled water [up to 100 mL]) was added to the supernatant. Then, 2 mL of potassium ferrocyanide solution (10.6% wt/vol in distilled water) was added to the 15-mL tubes containing the supernatant and zinc acetate solution. The tubes were centrifuged (7,168 × *g*, 10 min), and 1 mL of supernatant was transferred to another 15-mL tube, and 5 mL HCl solution (30% vol/vol in distilled water) was added. The tubes were incubated for 75 min at 28 to 30°C, after which OD_254_ measurements were taken. A standard curve (with concentrations of 0 to 1,000 μg mL^−1^ of GA) was created for calculating the micrograms of gibberellic acid per milliliter of the mixture (49, 54). This experiment was repeated twice, independently.

**1-aminocyclopropane-1-carboxylic acid (ACC) deaminase activity.** A culture-based assay was done for ACC deaminase activity as described previously (57). Bacterial isolates were spot-inoculated in three replicates and grown on Dworkin & Foster (DF) minimal salt medium (DF salts per L: 4.0 g KH_2_PO_4_, 6.0 g Na_2_HPO_4_, 0.2 g MgSO_4_·7H_2_O, 2.0 g glucose, 2.0 g gluconic acid, and 2.0 g citric acid with trace elements: 1 mg FeSO_4_·7H_2_O, 10 mg H_3_BO_3_, 11.19 mg MnSO_4_·H_2_O, 124.6 mg ZnSO_4_·7H_2_O, 78.22 mg CuSO_4_·5H_2_O, 10 mg MoO_3_, pH 7.2) supplemented with 3 mM ACC as the sole nitrogen source. Isolates that grew on the plates were able to produce ACC deaminase (57). This experiment was repeated three times, independently.

### Antifungal activity.

A culture-based assay against the fungal pathogen Magnaporthe grisea was done as described previously (24), with minor modifications. Briefly, each isolate was cultured in two parallel lines (2 to 2.5 cm apart from each other) on potato dextrose agar (PDA) medium. Magnaporthe grisea causing rice blast disease was isolated from leaves of rice plants grown in rooftop paddies outside the Schläppi lab and was spot cultured in the center of PDA plates (between two parallel bacterial lines, 0.5 to 1 cm away from either side). Control plates had the only fungus without bacteria. Plates were incubated at 28°C for 5 days. The radial growth of the fungus was examined to observe growth inhibition by each bacterium. The percentage of growth inhibition from three independent experiments was calculated and compared with the radial growth of fungus in control plates using the formula shown below with the following parameters: I, inhibition percentage; C, radial growth in control plate; T, radial growth in plates with bacterial isolates (24).

I = [(C – T)/C]  ×  100

**Lipase production.** An agar assay method was used to detect bacteria with lipase production ability. Luria-Bertani (LB) medium (tryptone [10 g], NaCl [10 g], yeast extract [5 g], agar [15 g]) was prepared in 490 mL of distilled water and supplemented with 1% Tween 20. Then, 2 μL of each isolated strain was spot-inoculated on the above-described medium in duplicate. One set of plates was incubated at 28°C for 3 to 4 days. Lipase production was detected by observing precipitation around colonies (61). This experiment was repeated three times, independently.

**Casein and gelatin hydrolyzing.** Protein hydrolysis was tested using both casein and gelatin media. Casein agar medium contained NB (Difco) with 1.5% (wt/vol) agar. Evaporated milk (50 mL) and 50 mL of sterile water were added to the sterile NB with agar (final volume = 1,000 mL), and 2 μL of each isolated strain (OD_600_ = 0.6) was spot-inoculated onto the medium in duplicate. Plates were incubated at 28°C for 24 h. Casein hydrolysis was examined by observing a halo around colonies (62). Gelatin medium contained 3 g L^−1^ beef extract, 5 g L^−1^ peptone, and 60 g L^−1^ gelatin in 1 L of distilled water. Bacterial solutions were stabbed into test tubes containing gelatin with inoculating needles. Tubes were incubated at 28°C for 2 weeks. Bacteria with gelatinase activity were able to liquify the medium (63). This experiment was repeated three times, independently.

**Siderophore production.** An agar assay method was used to detect bacteria producing siderophores that might help plants with iron uptake from the soil (63), as described previously (64). Specifically, chrome azurol S (CAS) agar was prepared by mixing four sterile solutions. Solution 1, the Fe-CAS indicator solution, contained 10 mL of 1 mM FeCl_3_·6H_2_O (in 10 mM HCl), 50 mL of an aqueous solution of CAS (1.21 mg mL^−1^), and 40 mL of an aqueous solution of hexadecyl-trimethylammonium bromide (HDTMA) (1.82 mg mL^−1^). Solution 2, the buffer solution, contained 30.24 g piperazine-*N,N*′-bis[2-ethanesulfonic acid] (PIPES) dissolved in 750 mL of salt solution (100 mL of MM9 salt solution dissolved in 750 mL of distilled water); 50 mL water was added to bring the final volume to 800 mL, and 1.5% (wt/vol) agar was added. To dissolve PIPES in the salt solution, 50% KOH was added until the pH of the solution was 6.8. Solution 3 contained 2 g glucose, 2 g mannitol, and trace elements (same ingredients mentioned in the IAA production method section) which were dissolved in 70 mL distilled water. Solution 4 contained 10% (wt/vol) Casamino Acids, which was filter-sterilized (0.2-μm membrane; Whatman syringe filters). All solutions, except solution 4, were autoclaved. After cooling to 50°C, solutions 3 and 4 were mixed, and then the buffer solution (solution 2) was added. The combined solutions were added to solution 1. The prepared medium (blue to dark green color) was poured into plates, and 2 μL of each isolated strain (OD_600_ = 0.6) was spot-inoculated onto the medium in duplicates. Plates were incubated at 30°C for 24 h. Siderophore production was examined by observing an orange halo around colonies (64, 65). This experiment was repeated three times, independently.

**Cellulase production.** The medium used for this test was the same as for lipase production, except that instead of Tween 20, the medium was supplemented with 1% carboxymethyl cellulose (CMC). A total of 2 μL of each isolated strain (OD_600_ = 0.6) (Escherichia coli as the negative control) was spot-inoculated onto the medium in duplicate. Plates were incubated at 28°C for 3 to 4 days. Cellulase production was detected through staining and destaining with 0.1% Congo red (wt/vol in distilled water) for 15 min and 1 M NaCl for 15 min, respectively (66). This experiment was repeated three times, independently.

**Ammonia (NH_3_) production.** A colorimetric assay was done following the methods of reference 66 with minor modifications. Freshly grown bacterial isolates were inoculated in 10 mL peptone water (15 g peptone water in 1,000 mL distilled water) and incubated at 28°C on a shaker (200 rpm) for 48 h. After incubation, 1 mL of each bacterial sample was transferred to a 1.5-mL tube and centrifuged for 10 min (2,218 × *g*) in a microcentrifuge. The supernatant was transferred to another 1.5-mL tube and 100 μL Nessler’s reagent (10% HgI_2_; 7% KI; 50% aqueous solution of NaOH [32%]) was added to each tube and incubated at room temperature for 30 min. Samples with the ability of ammonia production turned the color from yellow to orange. The optical density of each sample was measured in a UV-spectrophotometer at 520 nm. A standard curve (with concentrations of 0 to 300 μg mL^−1^ using ammonia carbonate) was created for calculating the micrograms of NH_3_ per milliliter of the mixture solution (66). This experiment was repeated three times, independently.

***In vitro* assay for salt tolerance.** Bacterial isolates were spot-inoculated in three replicates on lysogeny broth (LB) medium (tryptone [10 g L^−1^], NaCl [10 g L^−1^], yeast extract [5 g L^−1^], agar [15 g L^−1^]) supplemented with 0%, 2%, 4%, 6% and 8% NaCl. LB plates were incubated at 28°C for 5 days. Plates were examined for bacterial growth on LB with different NaCl concentrations (57). This experiment was repeated three times, independently.

**Whole-genome sequencing of 5 candidate PSB.** Five PSB isolates from the Carolino 164 (*indica*) endosphere, Carolino 164 phyllosphere, and Krasnodarskij 3352 (*japonica*) phyllosphere were chosen based on differing colony morphologies and their ability to produce plant hormones for whole-genome sequencing. Nucleic acid extraction of the 5 PSB was done using the Qiagen DNeasy blood and tissue extraction kit according to the manufacturer’s instructions. The DNA concentration of each sample was measured with a NanoDrop spectrophotometer. The PSB samples were then sent to the Microbial Genome Sequencing Center (www.migscenter.com) for whole-genome sequencing. Libraries were prepared as described previously (67) and sequenced on an Illumina NextSeq 550 instrument, yielding 151-bp paired-end reads.

**Sequence analysis and annotation of sequenced PSB.** Genome sequences of 5 PSB isolates were analyzed using KBase, and data were deposited into the public narrative site https://narrative.kbase.us/narrative/52526. Sequence reads were quality checked with FastQC v0.11.9 (68). Genomes were assembled with SPAdes v3.13.0 (69) and annotated with RASTtk (51). Initial genome relatedness was determined by inserting a genome into a phylogenetic tree with FastTree 2 (70) and calculating the average nucleotide identity with FastANI (71) and JSpeciesWS (26). Taxonomic classification was done with the Genome Taxonomy Database toolkit v1.6.0 (GTDB-tk) (72). A final maximum likelihood tree was generated using the GToTree v1.6.11 implementation of IQ-TREE 2 v2.1.4 (73) and its dependencies (74–79).

**Phenotypic fingerprint of sequenced PSB.** This test is specialized for characterizing bacteria phenotypically using the Biolog Gen III microplate. The 5 sequenced PSB were characterized utilizing 71 carbon sources and 23 chemical sensitivity assays on 96-well microplates (Fig. S2). Isolated bacteria were grown on NA medium and suspended in inoculating fluid (provided with the 96-well microplates) using sterile swabs. The turbidity of the inoculating fluid was 95%. Then, 100 μL of the bacterial suspension was added to each well, and the microplates were incubated at 28°C for 2 to 3 days. Wells that were positive for a specific carbon source turned purple due to formazan (if absorbance determined in a microplate reader exceeded that of the control well) or were sensitive to a chemical material (if formazan absorbance was less than that of the control well) (www.biolog.com). This experiment was repeated three times.

**Antibacterial activity of the sequenced PSB against each other.** Two culture-based antibacterial activity assays, overlay-agar, and cross-streak, were done to determine whether the 5 sequenced PSB interacted with each other:

**(i) Overlay-Agar assay.** Isolated bacteria were grown in nutrient broth (NB) medium at 30°C for 24 h. Freshly grown bacteria (OD_600_ = 0.6), were spot-inoculated on NA medium and incubated at 30°C for 48 h. After 2 days of incubation, plates were inverted under the hood over filter papers soaked in chloroform for 15 min. Plates were put upright without lids to let extra chloroform evaporate. Then, 10 mL of NB medium (containing 1.5% agar at 50°C) mixed with 200 μL of an indicator strain (the organism to be tested for susceptibility) was poured on the surface of NA plates and incubated at 30°C for 48 to 72 h. After incubation, inoculated bacteria were checked for zones of inhibition (80). This assay was repeated three times, independently.

**(ii) Cross-streak agar assay.** Isolated bacteria were grown in NB medium at 30°C for 24 h. Freshly grown bacteria (OD_600_ = 0.6) were inoculated in a single 1-cm-wide linear streak down the center of NA plates, and this process was repeated for each bacterium. Plates were incubated at 30°C for 48 h. The organism to be tested for susceptibility was streaked in lines perpendicular to the bacterium that was horizontally streaked (up to 1 mm). Plates were incubated at 30°C for 48 to 72 h. After incubation, plates were examined for zones of inhibition around the initial bacterium streak, and the width of each zone of inhibition was measured in millimeters (81). This assay was repeated three times, independently.

**Evaluating the influence of sequenced PSB on rice growth and development.** The influence of five sequenced PSB on rice growth and development was evaluated by seed inoculation with bacterial suspensions (control plants: seeds inoculated in bacterium-free KCl). Shoot and root lengths were measured when plants were 14 days old. Furthermore, when plants were 2 weeks old, the dry weight root/shoot ratio was determined to measure nutrient uptake ability (the higher the root/shoot ratio, the more nutrients are taken up by plants) (36).

**Shoot and root length.** Bacterial cells were harvested by centrifugation and rinsed with sterile water and then resuspended in 0.85% KCl (biological saline) (82). Carolino 164 and Kasalath (two rice accessions representing *indica* subspecies) as well as Krasnodarskij 3352 and Zhonghua 11 (two rice accessions representing *japonica* subspecies) seeds were surface-sterilized as described earlier and soaked in the bacterial suspension (10^8^ cell mL^−1^) overnight (83). Seeds soaked in KCl solution were used as controls and counted as non-bacterium-soaked seeds. Both soaked and non-bacterium-soaked seeds were germinated in 1× MS medium at 30°C for 2 days. Germinated seeds were transferred to autoclaved PCR strips placed in boxes filled with sterile water to grow hydroponically. The boxes were put in controlled chambers (12 h of light [28°C] and 12 h of dark cycle [24°C]) for 14 days). At day 10, the water in the boxes was replaced with ¼ Murashige-Skoog (MS) medium. Shoot and root lengths were measured when plants were 14 days old.

To confirm that bacteria were attached to the seeds during overnight shaking (the process of bacterial inoculation) and that uninoculated soaked seeds were free of bacteria, 5 rice seeds from each of the treatments (KCl or bacterial suspension in KCl) were placed onto nutrient agar (NA) plates and incubated at 30°C overnight. Inoculated seeds had bacterial colonies around them, while uninoculated seeds showed no growth of bacteria or contamination (Fig. S1). In addition, three inoculated and uninoculated seeds were transferred into 1 mL phosphate buffer solution (PBS) separately (1 seed per tube), and then tubes were sonicated in a water bath for 10 min (Branson; 50 to 60 Hz). Serial dilutions of the sonicated PBS containing seeds were prepared (from 10^−1^ to 10^−5^), and 10 μL of each dilution was dropped onto NA in triplicate. Plates were incubated at 30°C overnight. No growth was observed from tubes containing uninoculated seeds. From tubes containing inoculated seeds, the mean CFU per seed was calculated. The average CFU per seed for Kasnodarskij 3352 and Zhonghua 11 was 1.13 × 10^4^ and 2.03 × 10^4^, respectively. The average CFU per seed for Carolino 164 and Kasalath was 1.56 × 10^4^ and 1.78 × 10^4^, respectively (Fig. S1).

**Dry weight root/shoot ratio.** Shoots and roots of 15 inoculated and uninoculated plants (2-week-old plants) were isolated and put into a dry oven at 50°C for 48 h (83). After 2 days of incubation, the dry weight root/shoot ratio of each of 15 plants per trial (total of 3 trials) was measured to evaluate the ability of plants to take up nutrients while they were inoculated with bacteria.

### Data availability.

All sequence reads were deposited at the NCBI Sequence Read Archive (SRA) under BioProject no. PRJNA667792 and SRA accession no. SRS7484588 to SRS7484592. All assemblies and annotations can be viewed, manipulated, and downloaded from the open access KBase narrative at https://narrative.kbase.us/narrative/52526. We have also created a static narrative to support the article, which can be accessed here at https://kbase.us/n/52526/166/.

### Statistical analysis.

All statistical comparisons (ANOVA and Tukey *post hoc*) among the 25 PSB was done using Minitab statistical software v20.2.0.0 (free access from University of Wisconsin Milwaukee). Comparisons (Kruskal-Wallis, Dunn *post hoc*) between shoot and root lengths or dry weight root/shoot ratio were done using R v4.0.4. Kruskal-Wallis and Dunnett *post hoc* tests were implemented using the rstatix R package (84).
