# Supplementary material for: Plant Growth-Promoting Activity of Bacteria Isolated from Asian Rice (Oryza sativa L.) Depends on Rice Genotype
Source: Microbiol Spectr. 2022 Jul 11;10(4):e02787-21. doi: 10.1128/spectrum.02787-21 (PMC9431195; doi:10.1128/spectrum.02787-21)
Supplement: Supplemental file 1 — Supplemental material. Download spectrum.02787-21-s0001.pdf, PDF file, 0.9 MB [file spectrum.02787-21-s0001.pdf]

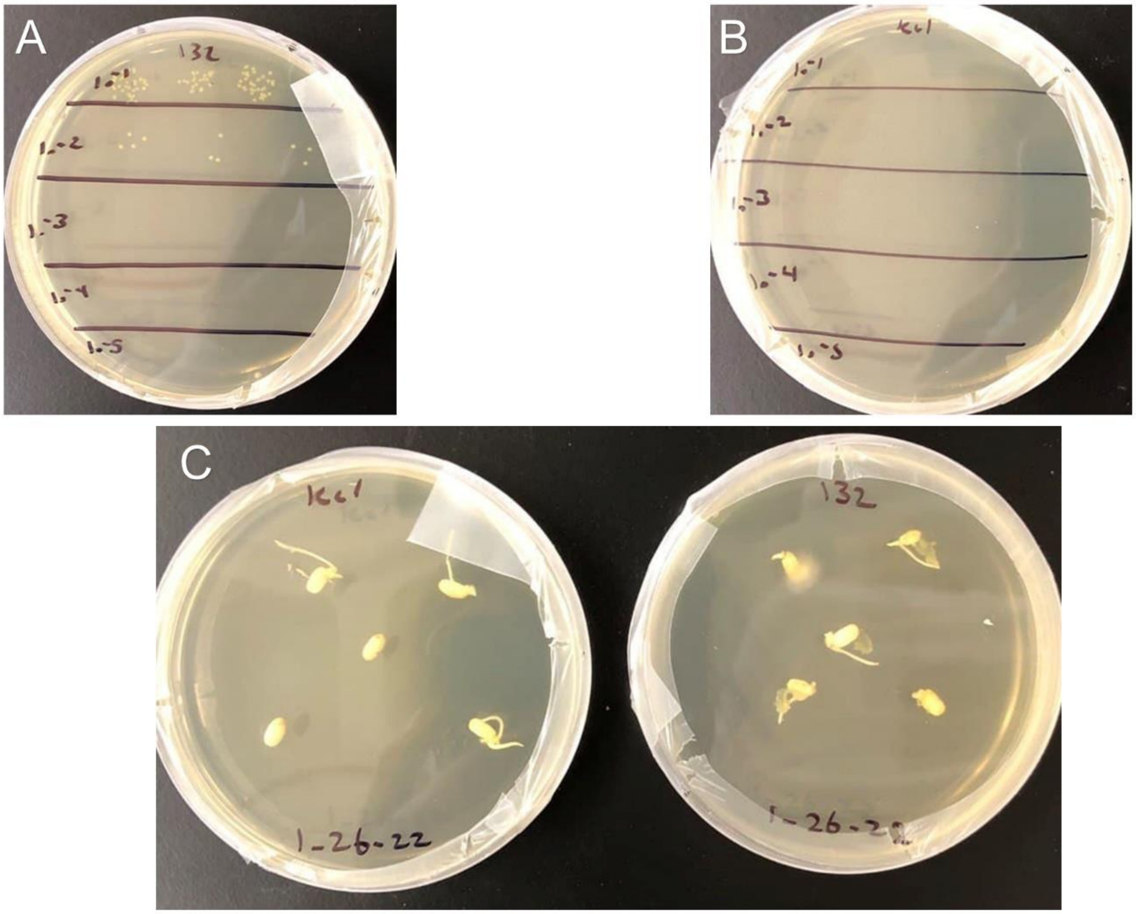

FIG S1 (A) Bacterial colonies from 10 µL drops (triplicates) of serial dilutions of 1 mL PBS containing 1 inoculated seed after sonication in three replicates. This experiment was done twice independently. The average CFU per seed for *japonica* was: 1.13 x 10^4. The average CFU per seed for *indica* was: 1.56 x 10^4. **(B)** No bacterial growth from seeds soaked in sterile KCl. **(C)** 5 seeds on NA medium after overnight shaking in KCl containing 10^8 bacterial cells (right) or cell-free KCl (left). Bacterial growth from soaked seeds in bacterial suspension can be seen, while no bacterial growth was observed in seeds soaked in cell-free KCl.

GEN III MicroPlate

A1 Negative Control	A2 Dextrin	A3 D-Maltose	A4 D-Trehalose	A5 D-Cellobiose	A6 Gentiobiose	A7 Sucrose	A8 D-Turanose	A9 Stachyose	A10 Positive Control	A11 pH 6	A12 pH 5
B1 D-Raffinose	B2 α-D-Lactose	B3 D-Melibiose	B4 β-Methyl-D-Glucoside	B5 D-Salicin	B6 N-Acetyl-D-Glucosamine	B7 N-Acetyl-β-D-Mannosamine	B8 N-Acetyl-D-Galactosamine	B9 N-Acetyl Neuraminic Acid	B10 1% NaCl	B11 4% NaCl	B12 8% NaCl
C1 α-D-Glucose	C2 D-Mannose	C3 D-Fructose	C4 D-Galactose	C5 3-Methyl Glucose	C6 D-Fucose	C7 L-Fucose	C8 L-Rhamnose	C9 Inosine	C10 1% Sodium Lactate	C11 Fusidic Acid	C12 D-Serine
D1 D-Sorbitol	D2 D-Mannitol	D3 D-Arabitol	D4 myo-Inositol	D5 Glycerol	D6 D-Glucose-6-PO4	D7 D-Fructose-6-PO4	D8 D-Aspartic Acid	D9 D-Serine	D10 Troleandomycin	D11 Rifamycin SV	D12 Minocycline
E1 Gelatin	E2 Glycyl-L-Proline	E3 L-Alanine	E4 L-Arginine	E5 L-Aspartic Acid	E6 L-Glutamic Acid	E7 L-Histidine	E8 L-Pyroglutamic Acid	E9 L-Serine	E10 Lincomycin	E11 Guanidine HCl	E12 Niaproof 4
F1 Pectin	F2 D-Galacturonic Acid	F3 L-Galactonic Acid Lactone	F4 D-Gluconic Acid	F5 D-Glucuronic Acid	F6 Glucuronamide	F7 Mucic Acid	F8 Quinic Acid	F9 D-Saccharic Acid	F10 Vancomycin	F11 Tetrazolium Violet	F12 Tetrazolium Blue
G1 p-Hydroxy-Phenylacetic Acid	G2 Methyl Pyruvate	G3 D-Lactic Acid Methyl Ester	G4 L-Lactic Acid	G5 Citric Acid	G6 α-Keto-Glutaric Acid	G7 D-Malic Acid	G8 L-Malic Acid	G9 Bromo-Succinic Acid	G10 Nalidixic Acid	G11 Lithium Chloride	G12 Potassium Tellurite
H1 Tween 40	H2 γ-Amino-Butryric Acid	H3 α-Hydroxy-Butyric Acid	H4 β-Hydroxy-D,L-Butyric Acid	H5 α-Keto-Butyric Acid	H6 Acetoacetic Acid	H7 Propionic Acid	H8 Acetic Acid	H9 Formic Acid	H10 Aztreonam	H11 Sodium Butyrate	H12 Sodium Bromate

FIG S2 Layout of 71 carbon sources and 23 chemical sensitivity assays (shaded) on the 96 well microplate.

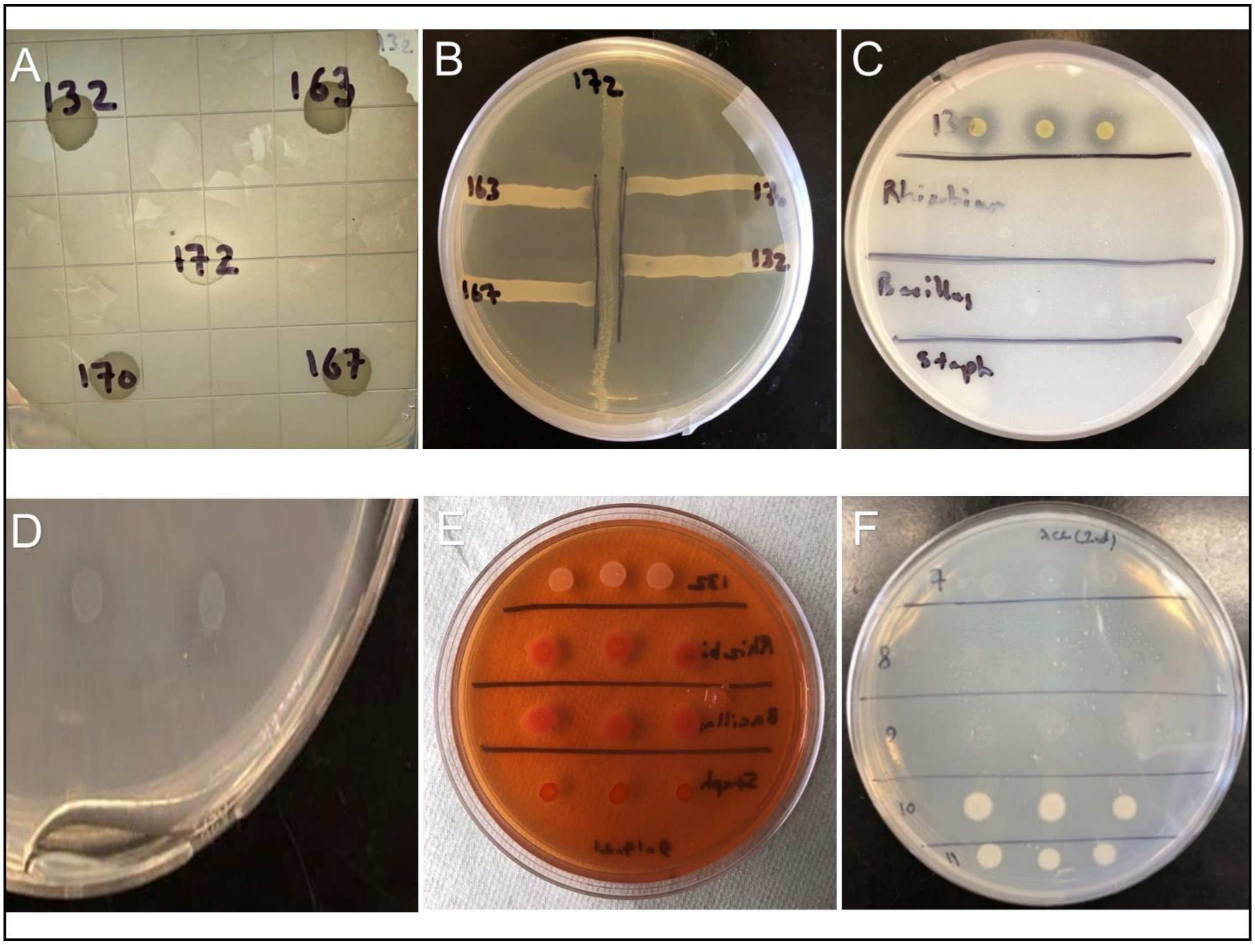

FIG S3 (A) Overlay agar assay. Halo around 172 (*Paenibacillus graminis*), showing growth inhibition when 132 (*Pseudomonas mosselii*) is overlaid. Halo around 172 was also observed, when the other three phosphate solubilizing bacteria (PSB) were overlaid: 163, *Paenibacillus rigui*; 167, *Brevibacillus sp900114075*; 170, *Microvirga sp003151255*. **(B)** Cross-streak assay. Growth inhibition of the other four PSB is shown when 172 is in the middle of the NB plate. **(C)** Bacterium 132 isolated from the *indica* phyllosphere has high phosphate solubilization ability as shown by strong halo formations (2.36 cm) around the bacterial colonies. *Rhizobacterium* sp., *Bacillus* sp., and *Staphylococcus aureus* are negative controls. **(D)** Bacterium n00078 isolated from *japonica* root endosphere has zinc solubilization ability as shown by halo formation around the bacterial colony. **(E)** Bacteria with nitrogen fixation ability shown on CR-YMA plates. The lower the absorption of Congo Red dye, the higher the ability for nitrogen fixation. In this figure, one strong nitrogen fixing bacterium (top 3 colonies; *P. mosselii*) is along with *Rhizobacterium* sp. and *Bacillus* sp., as positive controls and *Staphylococcus aureus* as a negative control. **(F)** Phosphate solubilizing bacteria n00007 (7), n00008 (8), n00009 (9), n00010 (10), and n00011 (11) are shown with the ability of ACC deaminase production based on their growth on DF minimal salt medium supplemented with 3 mM ACC as sole nitrogen source.

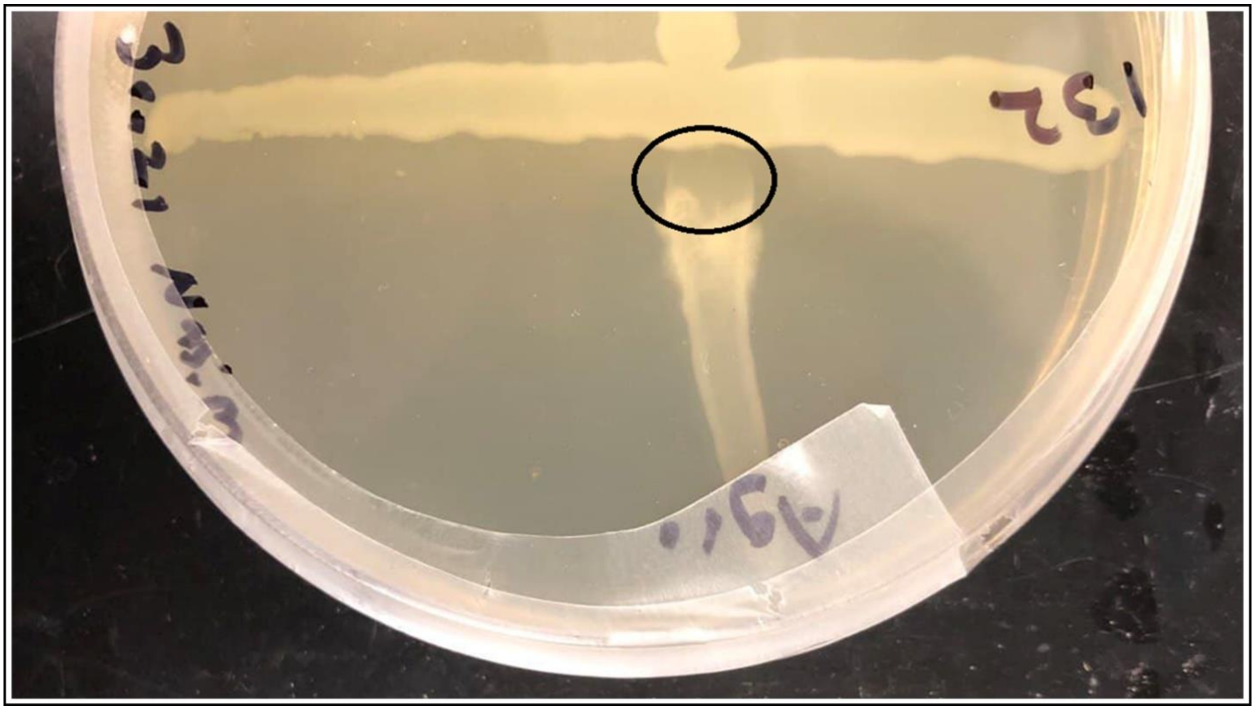

FIG S4 Cross-streak assay. Growth inhibition of the *Agrobacterium tumefaciens* is shown (circle) when 132 (*Pseudomonas mosselii*) is in the middle of the NB plate.

Table S1 List of two commercial soil contents that used for rice growth

Contents	Nature's care	Pro-Mix
Total Nitrogen (N)	0.09%	0.30%
Ammoniacal Nitrogen	0.008%	0.01%
Other water-soluble Nitrogen	0.028%	0.04%
Water insoluble Nitrogen	0.054%	0.25%
Available Phosphate (P_2O_5)	0.08%	0.10%
Soluble Potash (K_2O)	0.09%	0.10%
Calcium	0.02%	-
	Contains 0.054% slowly available Nitrogen (N) from poultry litter alfalfa meal and kelp meal	-

Table S2 List of culture-based plant growth promoting assays

Direct mechanisms of PGPB assays	Reference(s)
Zinc solubilization	(27)
Nitrogen fixation	(25, 26)
Indoleacetic acid (IAA) production (phytohormone)	(30)
Gibberellic acid production (phytohormone)	(28, 29)
1-aminocyclopropane-1-carboxylic acid (ACC) deaminase activity	(27)
**Indirect mechanisms of PGPB assays**	**Reference(s)**
Antifungal activity	(30, 31)
Lipase production	(31)
Casein and gelatin hydrolyzing (proteases)	(32, 33)
Cellulase production	(36)
Siderophore production	(34, 35)
Ammonia (NH_3) production	(39)

Table S3 Phosphate solubilizing bacteria with nitrogen fixation ability on CR-YMA plates. ++ (Pale white-pink), + (White-pink), - (Strong pink-red). Bacterial colonies with colors ranging from white to pale white-pink were able to fix nitrogen due to cleavage of the azo bond (-N=N-) in Congo Red.

PSB codes	Colony color
n00007	-
n00008	-
n00009	-
n00010	+
n00011	++
n00014-1	++
n00014-2	++

n00015	++
n00016	++
n00024-1	++
n00024-2	++
n00064	-
n00069	-
n00078	-
n00080	-
n00082	-
n00086	+
n00093	++
n00128	++
n00132	++
n00163	++
n00167	++
n00170	++
n00170-2	++
n00172	+
Rhizobium sp.	++
Bacillus sp.	++
Staphylococcus aureus	-

Table S4 Number of bacterial isolates from two accessions, Krasnodarskij 3352 and Carolino 164, representing the *japonica* and *indica* subspecies of rice (*Oryza sativa* L.), respectively.

**Rice subspecies/ plant tissue compartment**	**Root rhizoplane**	**Root endosphere**	**Phyllosphere**
japonica	36	30	17
indica	20	22	15

Table S5 Phenotypic characteristics of isolated bacteria from *indica* and *japonica* rhizoplane, endosphere, and phyllosphere.

***Indica* rhizoplane bacteria**	**Form**	**Elevation**	**Color**	**Size**	**Margin**	**Cell morphology**	**G+/G-**
n00001	Circular	Slightly raised	Yellow	M	Entire	Cocci	G+
n00002	Circular	Slightly raised	Milk white	M	Entire	Cocci	G+
n00003	Irregular	Flat	Milk white	L	Undulate	Rod-shape	G-
n00004	Circular	Slightly raised	Milk white	S	Entire	Cocci	G+
n00005	Circular	Slightly raised	Milk white	S	Entire	Cocci	G+

n00006	Circular	Slightly raised	Milk white	M	Entire	Cocci	G+
n00028	Circular	Raised	Milk white	M	Entire	Cocci	G+
n00029	Circular	Raised	Yellow	S	Entire	Cocci	G+
n00030	Circular	Raised	Milk white	M	Entire	Cocci	G+
n00031	Circular	Raised	Yellow	S	Entire	Cocci	G+
n00032	Circular	Flat	Milk white	S	Entire	Cocci	G+
n00033	Circular	Raised	Milk white	M	Entire	Rod-shape	G-
n00092	Circular	Raised	Yellow	L	Entire	Cocci	G+
n00093	Circular	Raised	White	S	Entire	Cocci	G+
n00094	Circular	Raised	Milk White	M	Entire	Rod-shape	G+
n00111	Circular	Raised	Yellow	S	Entire	Cocci	G+
n00112	Circular	Umbonate	White	L	Undulate	Rod-shape	G+
n00113	Circular	Raised	Yellow	S	Entire	Cocci	G+
n00115	Circular	Raised	Milk White	S	Entire	Cocci	G+
n00116	Circular	Raised	Yellow	S	Entire	Cocci	G+
***Indica* endosphere bacteria**	**Form**	**Elevation**	**Color**	**Size**	**Margin**	**Cell morphology**	**G+/G-**
n00012	Circular	Flat	Milk white	S	Slightly undulate	Cocci	G-
n00013	Circular	Slightly raised	Milk white	M	Undulate	Rod-shape	G+
n00014	Circular	Raised	Milk white	S	Filiform	Rod-shape	G+
n00015	Circular	Slightly raised	Milk white	M	Undulate	Cocci	G+
n00041	Circular	Slightly Raised	Milk white	M	Lobate	Rod-shape	G+
n00042	Circular	Slightly Raised	Milk white	L	Undulate	Rod-shape	G+
n00043	Circular	Flat	Milk white	M	Undulate	Cocci	G+
n00044	Circular	Raised	White	M	Lobate	Cocci	G+
n00044-1	Circular	Raised	White	S	Entire	Rod-shape	G+
n00044-2	Circular	Raised	Milk white	M	Undulate	Diplococci	G+
n00044-3	Circular	Raised	Milk white	M	Undulate	Rod-shape	G+
n00045	Circular	Flat	Milk white	M	Undulate	Cocci	G+
n00070	Irregular	Flat	Cream	L	Filiform	Rod-shape	G+
n00106	Circular	Raised	Yellow	M	Entire	Cocci	G+
n00107	Circular	Raised	Milk White	M	Entire	Cocci	G+
n00108	Circular	Raised	Yellow	S	Entire	Cocci	G+
n00129	Circular	Flat	Transparent	S	Entire	Cocci	G+
n00130	Circular	Slightly Raised	Milk White	S	Entire	Rod-shape	G+
n00131	Circular	Slightly Raised	Milk White	S	Entire	Cocci	G+
n00147	Circular	Flat	Milk White	M	Lobate	Rod-shape	G+
n00163	Circular	Raised	White	S	Entire	Rod-shape	G+

n00173	Irregular	Slightly Raised	Milk White	S	Lobate	Rod-shape	G+
***Indica* phyllosphere bacteria**	**Form**	**Elevation**	**Color**	**Size**	**Margin**	**Cell morphology**	**G+/G-**
n00022	Irregular	Raised	Milk white	M	Undulate	Rod-shape	G+
n00022-1	Irregular	Raised	Milk white	S	Undulate	Cocci	G+
n00023	Irregular	Raised	Milk white	M	Lobate	Rod-shape	G+
n00024	insufficient growth	-	Milk white	M	-	Cocci	G+
n00025	Irregular	Raised	Milk white	M	Undulate	Cocci	G+
n00025-1	Circular	Slightly raised	Milk white	S	Entire	Cocci	G-
n00048	Circular	Raised	Milk white	M	Lobate	Cocci	G+
n00049	Irregular	Slightly Raised	Milk white	L	Undulate	Chain Cocci	G+
n00087	Irregular	Flat	Cream	L	Filiform	Rod-shape	G+
n00109	Circular	Raised	Yellow	S	Entire	Diplo cocci	G+
n00132	Circular	Slightly Raised	Milk White	S	Undulate	Rod-shape	G-
n00157	Circular	Raised	White	M	Entire	Diplo cocci	G+
n00158	Circular	Umbonate	Milk White	M	Undulate	Rod-shape	G+
n00166	Circular	Flat	Milk White	S	Entire	Cocci	G+
n00167	Circular	Flat	White	S	Entire	Rod-shape	G+
***Japonica* rhizoplane bacteria**	**Form**	**Elevation**	**Color**	**Size**	**Margin**	**Cell morphology**	**G+/G-**
n00007	Irregular	Umbonate	Milk white	S	Filiform	Rod-shape	G-
n00008	Irregular	Slightly raised	Milk white	M	Undulate	Cocci	G+
n00009	Irregular	Flat	Milk white	M	Undulate	Rod-shape	G-
n00010	Circular	Slightly raised	Milk white	L	Entire	Cocci	G+
n00011	Circular	Slightly raised	Milk white	M	Filiform	Rod-shape	G-
n00034	Circular	Raised	Yellow	L	Entire	Cocci	G-
n00035	Circular	Raised	Milk white	S	Entire	Cocci	G+
n00036	Circular	Raised	Yellow	M	Entire	Cocci	G+
n00039	Circular	Raised	Yellow	M	Entire	Rod-shape	G+
n00056	Circular	Raised	Milk White	S	Entire	Rod-shape	G+
n00057	Circular	Raised	Milk White	M	Entire	Rod-shape	G+
n00058	Circular	Slightly Raised	Yellow	S	Entire	Rod-shape	G+
n00059	Circular	Slightly Raised	White	S	Entire	Rod-shape	G+
n00060	Circular	Slightly Raised	Milk White	S	Entire	Cocci	G+

n00061	Circular	Slightly Raised	Milk White	S	Entire	Rod-shape	G+
n00062	Circular	Crateriform	Red	M	Entire	Rod-shape	G+
n00063	Circular	Raised	Milk White	M	Entire	Rod-shape	G-
n00064	Circular	Flat	Milk White	L	Undulate	Rod-shape	G+
n00097	Circular	Slightly Raised	Milk White	S	Entire	Rod-shape	G+
n00098	Circular	Flat	Yellow	S	Entire	Chain cocci	G+
n00099	Circular	Slightly Raised	Milk White	S	Entire	Cocci	G+
n00101	Circular	Raised	White	M	Entire	Cocci	G+
n00102	Circular	Flat	Transparent	S	Entire	Cocci	G+
n00103	Circular	Raised	Milk White	M	Entire	Rod-shape	G+
n00104	Circular	Raised	Yellow	L	Entire	Cocci	G+
n00105	Circular	Raised	White	S	Entire	Cocci	G+
n00117	Circular	Flat	Red	S	Entire	Cocci	G+
n00121	Circular	Raised	Milk White	S	Entire	Cocci	G+
n00123	Circular	Raised	Milk White	S	Entire	Cocci	G+
n00124	Circular	Raised	Milk White	S	Entire	Rod-shape	G+
n00125	Circular	Raised	Yellow	M	Entire	Cocci	G+
n00126	Circular	Raised	Milk White	S	Entire	Cocci	G+
n00133	Circular	Raised	Milk White	M	Entire	Cocci	G+
n00134	Circular	Raised	Milk White	S	Entire	Cocci	G+
n00138	Circular	Raised	Milk White	S	Entire	Rod-shape	G+
n00139	Circular	Umbonate	Milk White	M	Entire	Cocci	G+
***Japonica* endosphere bacteria**	**Form**	**Elevation**	**Color**	**Size**	**Margin**	**Cell morphology**	**G+/G-**
n00016	Circular	Raised	Milk white	M	Entire	Rod-shape	G+
n00017	Circular	Raised	Milk white	M	Undulate	Cocci	G+
n00018	Circular	Raised	Milk white	S	Entire	Rod-shape	G-
n00019	Circular	Raised	Milk white	S	Entire	Cocci	G+
n00020	Circular	Raised	Milk white	S	Undulate	Cocci	G+
n00046	Circular	Raised	Yellow	L	Entire	Cocci	G+
n00047	Circular	Umbonate	Milk white	S	Entire	Cocci	G+
n00071	Circular	Raised	Milk White	M	Entire	Cocci	G-
n00072	Circular	Slightly Raised	Milk White	S	Entire	Cocci	G+
n00073	Circular	Flat	White	S	Entire	Cocci	G+
n00074	Circular	Umbonate	White	M	Undulate	Rod-shape	G+
n00075	Circular	Raised	Milk White	M	Undulate	Cocci	G+
n00076	Circular	Crateriform	Milk White	M	Entire	Rod-shape	G+
n00077	Circular	Flat	White	S	Entire	Rod-shape	G+
n00078	Circular	Flat	White	M	Filiform	Rod-shape	G+
n00079	Circular	Flat	Milk White	M	Entire	Rod-shape	G+
n00080	Circular	Flat	White	M	Entire	Rod-shape	G+

n00081	Circular	Flat	Yellow	S	Entire	Cocci	G+
n00082	Irregular	Flat	White	L	Undulate	Chain Cocci	G+
n00083	Irregular	Flat	Cream	L	Filiform	Rod-shape	G+
n00084	Irregular	Flat	Cream	L	Filiform	Chain Cocci	G+
n00150	Circular	Umbonate	White	L	Undulate	Chain cocci	G+
n00152	Circular	Flat	Creame	M	Undulate	Rod-shape	G+
n00153	Circular	Umbonate	White	S	Entire	Cocci	G+
n00154	Circular	Slightly raised	White	S	Entire	Cocci	G+
n00155	Circular	Flat	White	S	Undulate	Rod-shape	G+
n00156	Circular	Flat	White	S	Entire with transpare nt margin	Rod-shape	G+
n00164	Circular	Slightly raised	White	S	Entire	Rod-shape	G+
n00165	Circular	Raised	Milk White	S	Entire	Cocci	G+
n00174	Irregular	Slightly Raised	Milk White	S	Lobate	Rod-shape	G+
***Japonica* phyllosphere bacteria**	**Form**	**Elevation**	**Color**	**Siz e**	**Margin**	**Cell morpholog y**	**G+/G -**
n00026	Circular	Flat	Milk white	S	Entire	Cocci	G+
n00027	Circular	Slightly raised	Milk white	S	Filiform	Rod-shape	G+
n00050	Circular	Umbonate	Milk white	M	Undulate	Cocci	G-
n00051	Circular	Flat	Milk white	S	Undulate	Cocci	G+
n00052	Circular	Umbonate	Milk white	M	Entire	Rod-shape	G+
n00053	Irregular	Slightly Raised	Red	S	Undulate	Rod-shape	G+
n00054	Circular	Flat	Yellow	S	Entire	Rod-shape	G+
n00055	Circular	Flat	Milk white	S	Undulate	Rod-shape	G-
n00088	Circular	Flat	Milk White	M	Entire with transpare nt margins	Cocci	G+
n00089	Circular	Flat	Milk White	M	Entire with transpare nt margins	Chain Cocci	G+
n00162	Circular	Raised	Yellow	S	Entire	Diplo cocci	G+
n00169	Circular	Raised	White	S	Entire	Rod-shape	G+
n00170	Circular	Raised	Milk White	S	Entire	Rod-shape	G-
n00170-1	Irregular	Raised	Milk White	M	Undulate	Rod-shape	G+
n00170-2	Circular	Raised	Milk White	M	Entire	Rod-shape	G+
n00171	Circular	Raised	Yellow	S	Entire	Diplo cocci	G+
n00172	Circular	Flat	White	S	Entire	Rod-shape	G+
n00175	Circular	Raised	Yellow	S	Entire	Diplo cocci	G+

Table S6 Indirect plant growth promoting assays and *in vitro* assay results for phosphate solubilizing bacteria (PSB) salt tolerance. "+" results are shaded.

PSB codes	Siderophore	Lipase	Cellulase	Antifungal activity (%)	Gelatin	Casein	Ammonia (μg/mL)	LB (2%) NaCl	LB (4%) NaCl	LB (6%) NaCl	LB (8%) NaCl
n00007	+	+	-	23.3	+	-	780.24	+	+	-	-
n00008	-	+	+	4.43	+	-	811.58	+	+	-	-
n00009	+	+	-	25.14	+	-	850.20	+	+	-	-
n00010	+	+	-	20.9	-	-	805.08	+	+	-	-
n00011	+	+	+	21.82	+	+	929.22	+	+	-	-
n00014-1	+	+	+	22.67	Slight +	-	895.55	+	+	+	-
n00014-2	+	+	+	19.76	-	-	894.48	+	-	-	-
n00015	+	+	+	25.74	+	-	883.17	+	-	-	-
n00016	+	+	+	11.17	-	-	859.05	+	-	-	-
n00024-1	+	+	+	14.98	-	-	871.64	+	-	-	-
n00024-2	+	+	+	18.88	-	-	835.04	+	-	-	-
n00064	+	+	-	7.06	+	-	828.38	+	+	-	-
n00069	+	-	Slight +	8.77	-	-	858.33	+	+	-	-
n00078	-	-	Slight +	8.19	+	-	891.15	+	+	-	-
n00080	-	-	Slight +	2.68	Slight +	-	862.91	+	+	-	-
n00082	-	-	Slight +	0	Slight +	-	850.93	+	+	-	-
n00086	-	-	Slight +	0	-	-	840.47	+	+	-	-
n00093	-	-	Slight +	1.7543	-	-	876.94	+	+	-	-
n00128	+	+	+	23.73	+	-	846.75	+	+	-	-
n00132	+	+	+	5.75	+	+	811.10	+	+	-	-
n00163	+	+	+	0	+	-	803.43	+	+	-	-
n00167	+	+	+	3.95	Slight +	-	845.71	+	+	-	-
n00170	-	-	Slight +	3.74	-	-	823.14	+	+	-	-

n00170-2	+	+	+	22	-	-	856.44	+	+	-	-
n00172	-	-	Slight +	0	-	-	807.13	+	+	-	-

Table S7 Phosphate solubilizing bacteria (PSB) with ACC deaminase production. “Strong +” indicates PSB grew strongly on DF minimal salt medium supplemented with 3 mM ACC as sole nitrogen source, while “Slight +” indicates weak growth. “None” means no growth.

**PSB codes**	**ACC deaminase (overall 3 trials)**
n00007	Slight +
n00008	None
n00009	Slight +
n00010	Strong +
n00011	Strong +
n00014-1	Strong +
n00014-2	Strong +
n00015	Strong +
n00016	Strong +
n00024-1	Strong +
n00024-2	Strong +
n00064	Slight +
n00069	Slight +
n00078	None
n00080	None
n00082	Slight +
n00086	Slight +
n00093	None
n00128	Strong +
n00132	Slight +
n00170	Slight +
n00170-2	Strong +
n00172	Slight +
n00163	Slight +
n00167	Slight +
E. coli strain OP50	None
Agrobacterium	Strong +
Serratia marcescens	Slight +

Table S8 Bacterial phenotypic fingerprint of carbon source usage (Biolog microplates). “+” (shaded area) indicates bacterial use of the specific carbon source and “-” indicates that bacteria did not grow in the microplate. n00132, *Pseudomonas mosselii*; n00163, *Paenibacillus rigui*; n00167, *Brevibacillus sp900114075*; n00170, *Microvirga sp003151255*; n00172, *Paenibacillus graminis.*

Carbon Sources	*Agro*	n00132	n00163	n00167	n00170	n00172
Dextrin	+	+	+	+	+	+
D-Maltose	+	+	+	+	+	+
D-Trehalose	+	+	+	+	+	+
D-Cellobiose	+	+	+	+	+	+
Gentiobiose	+	+	+	+	+	+
Sucrose	+	+	+	+	+	+
D-Turanose	+	+	+	+	+	+
Stachyose	+	-	-	-	-	+
D-Raffinose	+	-	-	-	-	-
α-D-Lactose	+	-	-	-	+	+
D-Melibiose	+	-	-	-	-	+
ß-Methyl-D-Glucoside	+	+	+	+	+	+
D-Salicin	+	+	+	+	+	+
N-Acetyl-D-Glucosamine	+	+	+	+	+	+
N-Acetyl-ß-D-Mannosamine	+	+	+	+	+	+
α-D-Glucose	+	+	+	+	+	+
D-Mannose	+	+	+	+	+	+
D-Fructose	+	+	+	+	+	+
D-Galactose	+	+	+	+	+	+
D-Fucose	+	-	-	-	-	-
L-Fucose	+	-	-	-	-	-
L-Rhamnose	+	-	-	-	-	-
Inosine	+	+	+	+	+	+
D-Sorbitol	+					
D-Mannitol	+	+	+	+	+	+
D-Arabitol	+	-	-	-	-	-
myo-Inositol	+	-	-	-	-	-
Glycerol	+	-	+	+	+	+
D-Glucose-6-PO_4	+	-	-	-	-	-
D-Fructose-6-PO_4	+	-	-	-	-	-
D-Aspartic Acid	+	-	-	-	-	-
L-Alanine	+	-	-	-	-	-

**L-Aspartic Acid**	+	-	-	-	-	-
**L-Glutamic Acid**	+	-	-	-	-	-
**L-Pyroglutamic Acid**	+	-	-	-	-	-
**L-Serine**	+	-	-	-	-	-
**Pectin**	+	+	+	+	+	+
**D-Galacturonic Acid**	+	-	-	-	-	-
**L-Galactonic Acid Lactone**	+	-	-	-	-	-
**D-Gluconic Acid**	+	+	+	+	+	+
**D-Glucuronic Acid**	+	-	-	-	-	-
**Glucuronamide**	+	-	-	-	-	-
**Mucic Acid**	+	-	-	-	-	-
**Quinic Acid**	+	-	-	-	-	-
**Methyl Pyruvate**	+	-	+	+	+	-
**D-Lactic Acid Methyl Ester**	+	-	-	-	-	-
**L-Lactic Acid**	+	+	+	+	+	+
**D-Malic Acid**	+	-	-	-	-	-
**L-Malic Acid**	+	-	-	-	-	-
**Bromo-Succinic Acid**	+	-	-	-	-	-
**Tween 40**	+	+	+	+	+	+
**Acetoacetic Acid**	+	+	+	+	+	
**Propionic Acid**	+	-	-	-	-	-
**Acetic Acid**	+	-	-	-	-	-

Table S9 Bacterial phenotypic fingerprint of chemical sensitivity (Biolog microplates). "+" (shaded area) indicates bacterial resistance to a specific chemical and "-" indicates bacterial sensitivity to a specific chemical.

Chemical inhibitors resistance	*Agro*	n00132	n00163	n00167	n00170	n00172
pH 6	+	+	+	+	+	-
1% NaCl	+	+	+	+	+	+
1% Sodium Lactate	+	+	+	+	+	+
Troleandomycin	+	-	-	-	-	-
Rifamycin SV	+	-	-	-	-	+
Lincomycin	+	-	-	-	-	
Guanidine HCl	-	+	+	+	+	+
Tetrazolium Blue	-	-	-	-	-	-
Nalidixic Acid	-	+	+	+	+	-
Lithium Chloride	-	+	+	+	+	+
Potassium Tellurite	+	+	+	+	+	-
Aztreonam	+	+	+	+	+	-
Sodium Butyrate	-	-	-	-	-	+

Table S10 Growth inhibition by n00172 (*Paenibacillus graminis*). Two antibacterial assays, overlay-agar and cross streak, show n00172's ability to inhibit the growth of the four other phosphate solubilizing bacteria (PSB). In overlay-agar test, "+" (shaded area) indicates presence of an inhibition halo around n00172 when each of these four PSB are overlaid. In the cross-streak test, growth inhibition of the four other PSB when crossing the n00172 bacterium is shown in mm (the test was done twice). n00132, *Pseudomonas mosselii*; n00163, *Paenibacillus rigui*; n00167, *Brevibacillus sp900114075*; n00170, *Microvirga sp003151255*.

Antibacterial activity/Bacteria	n00132	n00163	n00167	n00170	n00172
Overlay-agar	-	-	-	-	+
Cross-streak	2 mm/1 mm	3 mm/1 mm	1 mm/1 mm	1 mm/1 mm	-

Table S11 Secondary metabolite regions of isolate n00172 (*Paenibacillus graminins*) identified using strictness 'relaxed'. NRPS = Non-ribosomal peptide synthetase cluster, Type I PKS = Type I Polyketide synthase.

Region	Type	From	To	Most similar known cluster	Similarity
3.1	NPRS, T1PKS, NRPS-like	181,635	238,230		
4.1	cyclic-lactone-autoinducer	812	21,351		
4.2	RRE-containing	55,265	76,665		
5.1	cyclic-lactone-autoinducer	34,965	55,683		
7.1	NPRS, T1PKS	1	38,457	xenocoumacin 1 / xenocoumacin II, NRP + Polyketide:Modular type I	28%
10.1	NPRS, transAT-PKS	154,535	193,126		
13.1	cyclic-lactone-autoinducer	150,188	161,901		
20.1	NPRS, transAT-PKS, T1PKS, NRPS-like	1	97,214		
30.1	lassopeptide	17,331	41,158	Paeninodin, RiPP	100%
33.1	RRE-containing	25,743	47,170		
51.1	cyclic-lactone-autoinducer	4,648	25,296		
55.1	cyclic-lactone-autoinducer	16,231	36,755		
69.1	NRPS-like	1	13,082		
71.1	NPRS	1	10,121		
76.1	transAT-PKS-like	1	4,361		
77.1	NPRS	1	2,877		
83.1	NPRS	1	1,397		
